# Oral Nanomicelle‐Mediated Sequential Butyrate Production in the Intestine for Ulcerative Colitis and Immunosuppression Treatment

**DOI:** 10.1002/advs.77454

**Published:** 2026-08-27

**Authors:** Feifei Xin, Zhike Xie, Danyue Zhao, Lin Yang, Yiqun Wan, Zuwei Liu, Situ Xiong, Ying Zhang, Lei Feng, Hao Wan

**Affiliations:** ^1^ State Key Laboratory of Food Science and Resources Nanchang University Nanchang Jiangxi People's Republic of China; ^2^ Department of Food Science and Nutrition The Hong Kong Polytechnic University Hong Kong People's Republic of China; ^3^ Research Institute for Future Food The Hong Kong Polytechnic University Hong Kong People's Republic of China; ^4^ Department of Urology The First Affiliated Hospital Jiangxi Medical College Nanchang University Nanchang Jiangxi People's Republic of China

**Keywords:** butyrate, immunosuppression, nanomicelle, self‐assembly, ulcerative colitis

## Abstract

Butyrate (BA) is essential for intestinal health, but its intestinal therapeutic applications are hindered by its malodorous nature, premature gastric absorption, and rapid intestinal metabolism. To address these challenges, an inodorous macromolecule β‐glucan‐BA (GLU‐BA) is synthesized via one‐step esterification, allowing for BA production in the intestine with burst yet gradual dynamics. After oral administration, GLU‐BA self‐assembles into nanomicelles, preventing premature gastric absorption. Upon reaching the intestine, endogenous esterases rapidly hydrolyze its ester bonds, triggering a burst BA release while exposing GLU, with the GLU subsequently metabolized by gut microbiota to gradually generate BA. Such sequential production enabled by a single oral administration of GLU‐BA ensures significant intestinal BA availability as early as 0.5 h post administration, lasting for at least 12 h. This leads to co‐achievement of prompt intervention and sustained therapeutic efficacy, as demonstrated in mouse models of ulcerative colitis and immunosuppression. Additionally, GLU‐BA restores gut microbiota dysbiosis, during which BA‐producing bacteria are enriched, reinforcing BA intestinal replenishment. With excellent biocompatibility (negligible toxicity after 366 days of daily oral administration), scalable synthesis (∼54 g per one‐pot reaction), and broad‐spectrum bioactivities of BA, GLU‐BA demonstrates to be a promising candidate for clinical applications in treating diseases associated with intestinal disorders.

## Graphical abstract

After oral administration, inodorous GLU‐BA self‐assembles into nanomicelles in aqueous environments to resist premature gastric adsorption. Upon reaching the intestine, endogenous esterases rapidly cleave the built‐in ester bonds in these nanomicelles to release BA and GLU, with GLU further metabolized by gut microbiota to gradually generate BA, enabling burst yet gradual BA production in the intestine for disease treatment.

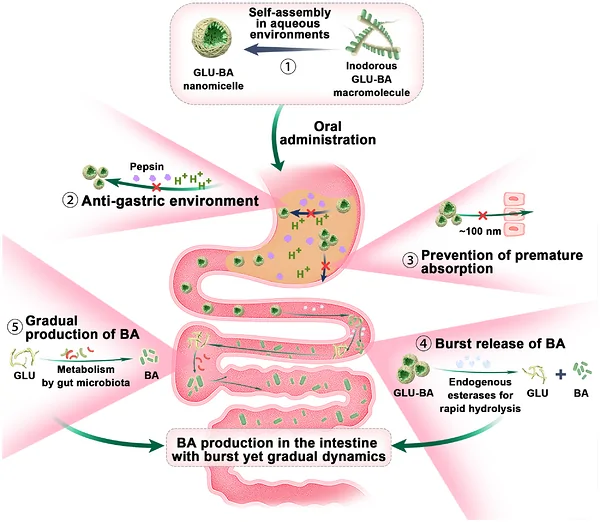

## 1 Introduction

With the progression of multi‐omics, gut microbiota has been deciphered to play a crucial role in various physiological activities, primarily through the production of metabolites that impact the host [1, 2, 3]. Among these metabolites, butyrate (BA), a type of short‐chain fatty acids (SCFAs), has received considerable attention owing to its ability to maintain intestinal homestasis [4], implying the potential of BA as a versatile therapeutic for the intervention of diseases associated with intestinal dysfunction. However, it is not practical to consume BA directly as it has a foul, long‐lasting smell and taste [5]. Because BA is readily absorbed in the upper gastrointestinal tract, its therapeutic effects in the intestine are severely compromised [6]. Notably, even with the addressing of foul smell/taste and premature gastric absorption by some strategies [5, 7], the rapid metabolism of BA as an energy source by colonocytes still limits its ability to exert pharmacological actions over a prolonged period. In this context, direct administration of high concentrations of BA into rectum was proposed to maintain sufficient BA in the intestine, which has been successfully implemented in the clinical treatment of ulcerative colitis (UC) [8]. However, this strategy is invasive and incompatible with daily lifestyles. Therefore, a more controlled and practical dosing strategy is urgently needed to improve the therapeutic benefits of BA in the intestine.

Targeted supplementation of BA in the intestine via oral formulations has emerged as an effective strategy. Unfortunately, almost all oral formulations for BA intestinal supplementation have adopted either a burst‐release [5, 7, 9, 10] or a gradual‐liberation mode [11, 12], inevitably facing the challenges of either rapid intestinal metabolism or delayed therapeutic responses. This creates difficulty in achieving a balance between prompt intervention and sustained therapeutic efficacy. In addition, these BA oral formulations have incorporated non‐degradable exogenous synthetic polymers [13] or inorganic nanoparticles [14, 15, 16], which are cumbersome to prepare and pose potential biosafety concerns, greatly hindering clinical translation.

As a major class of prebiotics, natural polysaccharides (derived from natural resources such as plants, animals, and fungi) are a category of biopolymers that are crucial for life activities and are gaining increasing attention in the field of drug delivery owing to their excellent biocompatibility and biodegradability [17, 18]. Most polysaccharides with intrinsic polymer structures and spatial dimensions are poorly absorbed in the upper gastrointestinal tract [19], rendering them ideal candidates to construct oral formulations for intestinal delivery. Inspired by this, we hypothesized that forming covalent bonds that rapidly and selectively respond to the intestinal environment between polysaccharides and BA moieties would enable a targeted burst release of BA in the intestine, thereby facilitating rapid pharmacological action for prompt disease intervention. Worth noting, it has been reported that certain polysaccharides, such as β‐glucan (GLU, a polysaccharide approved by the U.S. Food and Drug Administration [FDA] as a food supplement [20]), can be metabolized by gut microbiota to gradually produce BA [21], based on which an oral formulation holds the potential to sustainably supplement BA in the intestine. This process would compensate for the rapid intestinal metabolism of BA, thereby reinforcing its sustained therapeutic efficacy.

In this study, we covalently attached the BA moiety to GLU to obtain an inodorous macromolecule (GLU‐BA) via a simple esterification reaction in one step (Figure 1a). GLU‐BA can self‐assemble into nanomicelles in the physiological environment due to its amphiphilic characteristics (i.e., hydrophilicity of GLU and hydrophobicity of the alkane chains of BA). Following oral administration, GLU‐BA nanomicelles remained stable while traversing the upper digestive tract, thereby avoiding premature gastric adsorption. Subsequently, they were rapidly broken down in the intestine via enzymatic hydrolysis of built‐in ester bonds by endogenous esterases (Figure 1b). Such a process contributed to the burst release of BA and exposure of GLU for gradual metabolism by the gut microbiota, leading to the sequential production of BA in the intestine with burst yet gradual dynamics. Consequently, the prompt intervention and sustained therapeutic efficacy for diseases associated with intestinal dysfunction was achieved, establishing GLU‐BA nanomicelles as effective oral therapeutics. This was demonstrated by relieving dextrose sodium sulfate (DSS)‐induced UC as well as by ameliorating cyclophosphamide (Cy)‐induced immunosuppression in mice through the restoration of damaged intestinal barriers and the rebalance of gut microbiota dysbiosis. Notably, we found that GLU‐BA nanomicelles also regulated the gut microbiota to enrich BA‐producing bacteria, such as *Clostridium leptum*, demonstrating to be an endogenous source for further advancing BA intestinal replenishment. Due to the excellent biocompatibility (negligible biotoxicity observed even after consecutive oral administration on a daily basis for 1 year), easily‐scalable synthesis, and broad‐spectrum bioactivities of BA [22, 23, 24, 25], the oral nanomicelles developed here hold great potential to be generalized to deal with various diseases, and potentially serve as preventive agents integrated into daily health regimens.

**FIGURE 1:**
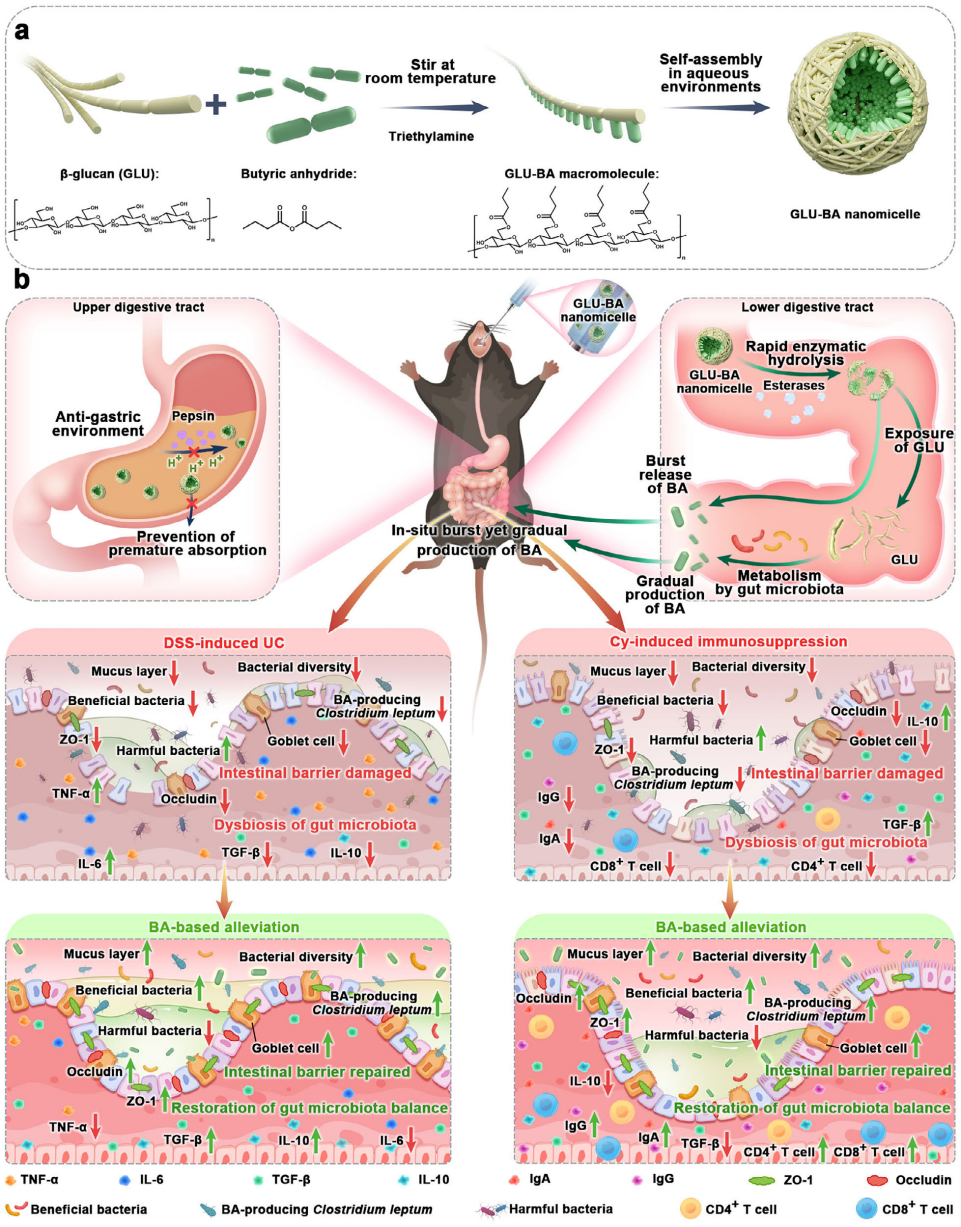
Schematic illustration of the synthesis of GLU‐BA and its therapeutic process via oral administration. (a) Preparation process of GLU‐BA. (b) Illustration depicting how GLU‐BA nanomicelles exerted therapeutic effects after oral administration. Following oral administration, GLU‐BA self‐assembled into nanomicelles to avoid premature absorption by the stomach, remaining intact and stable. Once GLU‐BA nanomicelles reached the intestine, their built‐in ester bonds were rapidly hydrolyzed by endogenous esterases to release BA and expose GLU in a burst manner, the latter of which was further metabolized by gut microbiota to gradually produce BA. Such a sequential in situ BA production behavior achieved prompt intervention and sustained therapeutic efficacy to alleviate DSS‐induced UC and Cy‐induced immunosuppression in mice.

## 2 Results and Discussion

### 2.1 Metabolomics Data Deciphering the Crucial Implication of BA in Maintaining Intestinal Homeostasis

UC, a typical disease closely associated with intestinal dysfunction, was first chosen for analysis to probe the potential of BA as a therapeutic agent. Through extensive search, we acquired different sets of intestinal metabolomic data for UC patients and healthy individuals from previous studies (Table S1). By comparing, it was found that the levels of BA were significantly decreased in UC patients compared to those in healthy individuals (Table S1), implying the crucial implication of BA in maintaining intestinal homeostasis, which has also been documented in other research [22, 25]. To further confirm this assumption, we modelled UC in mice (Figure 2a–c; Figures S1–S3) by DSS treatment, as described in our previous study [26], and compared the intestinal contents between UC and normal mice (sample quality control [QC] ≥ 99.86%, Figure S4). Principal component analysis (PCA) revealed a distinct pattern separation between the UC and normal mice (Figure 2d), reflecting the significant difference in their metabolic profiles. Based on these observations, differential metabolite analyses were performed. Compared to normal mice, 356 metabolites were up‐regulated, whereas 1130 metabolites were down‐regulated in UC mice (Figure 2e). In particular, among these down‐regulated metabolites, the reduced level of BA was found to be significant (Figure 2f; *p* < 0.0001), highlighting its crucial role in maintaining gut homeostasis.

**FIGURE 2:**
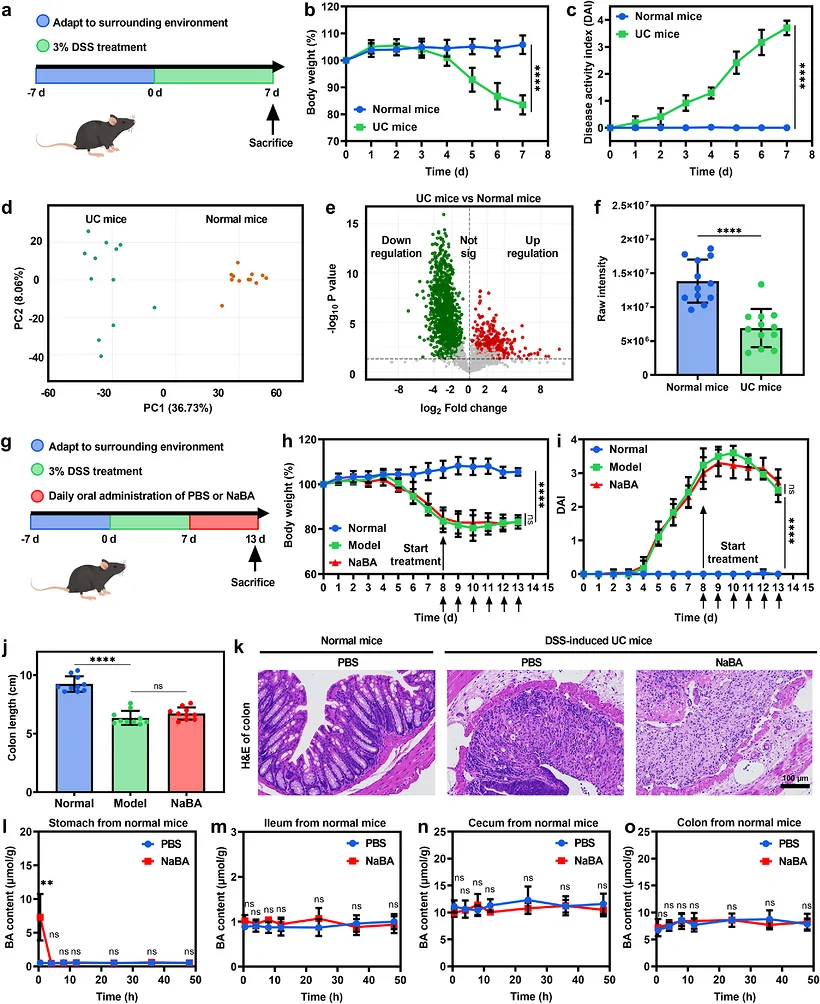
Implication of BA in maintaining gut homeostasis and evaluation of the therapeutic efficacy brought by direct oral administration of BA. (a) C57BL/6J mice were given drinking water containing 3% DSS continuously for 7 consecutive days to establish the mouse UC model. (b) Changes in the body weight of normal and UC mice over time, *n* = 12 per group. (c) Changes in the disease activity index (DAI) of normal and UC mice over time, *n* = 12 per group. (d) PCA of intestinal contents between normal and UC mice. *n* = 12 per group. (e) Volcanic plot of differential metabolites between normal and UC mice. *n* = 12 per group. (f) BA contents in normal and UC mice. *n* = 12 per group. (g) C57BL/6J mice were given drinking water containing 3% DSS for 7 consecutive days to establish the mouse UC model. Afterward, phosphate buffered saline (PBS) or NaBA was orally administered once daily for 6 consecutive days. (h) Changes in the body weight of mice over time among different groups, *n* = 10 per group. (i) Changes in the DAI of mice over time among different groups, *n* = 10 per group. (j) Quantitative analysis of colon lengths of mice from different groups at the end of intervention, *n* = 10 per group. (k) Representative hematoxylin & eosin (H&E) staining images of colon tissues of mice from different groups. (l‐o) In vivo BA levels in the stomach (l), ileum (m), cecum (n), and colon (o) after oral administration of PBS or NaBA to normal mice, *n* = 5 per group. Statistical analyses were performed using Student's t‐test and one‐way analysis of variance (ANOVA). ns represents no significance. **< 0.01 and ****< 0.0001 represent different statistical significances.

Hinted at by the above findings, it was proposed that direct oral replenishment of BA could be a straightforward approach to alleviating UC, despite its malodorous smell and taste. However, after administering sodium butyrate (NaBA) to UC mice (Figure 2g), we did not observe any sign of disease alleviation (Figure 2h–k; Figure S5). Using the validated gas chromatography (GC) method (Table S2), we further quantified BA concentrations in different gastrointestinal segments after oral administration. We regrettably found that this strategy resulted in considerable BA accumulation only in the stomachs of normal mice within 0.5 h post administration (Figure 2l; *p* = 0.0023), while negligible replenishment was observed in the intestines (Figure 2m–o) at all detected time points. Similar results were observed in DSS‐induced UC mice. NaBA administration significantly increased BA levels in the stomach at 0.5 h after gavage, whereas no significant elevation was detected in the intestinal segments at 12 h after administration (Figure S6). These findings were likely associated with premature upper gastrointestinal absorption and insufficient intestinal retention of BA, which severely compromised its sustained therapeutic utility, thus underscoring the essential for a more controlled supplementation strategy.

### 2.2 Synthesis and Characterization of GLU‐BA

In order to achieve efficient intestinal supplementation of BA via oral administration for therapeutic purposes, we designed GLU‐BA. To obtain GLU‐BA, an esterification reaction was initiated between the anhydride bond in butyric anhydride and the hydroxyl group in GLU to attach the BA moiety onto the polysaccharide chain by simply stirring at room temperature (Figure S7), for which systematic optimizations and characterizations were performed. Specifically, we synthesized a series of GLU‐BAs by adjusting the molar ratio of GLU to butyric anhydride (1:1, 1:2, 1:3, 1:4, 1:5, and 1:6). According to Fourier transform infrared spectroscopy (FTIR) analysis, all the resulting GLU‐BA samples exhibited distinct peaks within 1765–1725 cm^−1^ corresponding to the stretching vibration of the C = O in the ester bond [27], confirming the covalent attachment of the BA moiety to GLU (Figure 3a). To maximize the presence of the BA moiety in GLU‐BA, which is beneficial for BA‐mediated therapeutic processes, we chose GLU‐BA fabricated at a GLU‐to‐BA molar ratio of 1:2 for subsequent studies, considering the almost saturated attachment of BA indicated by zeta potential measurements (attachment rates reaching as high as 44.4%, Figure 3b). In view of the simplicity, the reaction can be easily scaled up, with a one‐pot reaction leading to as high as ∼54 g of GLU‐BA (Figure 3c). This quantity is sufficient to treat approximately 18 000 UC or immunosuppressive mice, as calculated based on the applied dosage in the subsequent therapeutic study. The yield could be further theoretically enhanced by simply increasing the amounts of ingredients added, making GLU‐BA suitable for industrial translation. Seen from nuclear magnetic resonance (NMR) results (Figure 3d; Figures S8 and S9), it can be clearly observed that new peaks between 0–2 ppm corresponding to saturated alkane chains of BA appeared in the ^1^H spectrum of GLU‐BA, further verifying the successful grafting of the BA moiety onto GLU. This led to the increase of the molecular weight from 482 kDa to 696 kDa, as measured by high‐performance gel permeation chromatography (HPGPC; Figure 3e; see Figure S10, for the calculation formula). GLU‐BA could be well dispersed in PBS to obtain a clear and transparent solution without a foul odor, suitable for oral administration (Figure 3f). As indicated by the dynamic light scattering (DLS) measurement (Figure 3g), GLU‐BA self‐assembled into a nanoparticle‐like structure with a narrow hydrodynamic size distribution in PBS. This was due to the amphiphilic property of GLU‐BA contributed by the hydrophilic GLU and hydrophobic BA segments, leading to the formation of nanomicelles. Transmission electron microscopy (TEM) images clearly identified the nanomicelle structure of GLU‐BA in the aqueous environment, with a maximum size of approximately 100 nm (Figure 3h) to avoid the premature absorption the stomach before reaching the intestine [28]. Eventually, GLU‐BA remained stable in PBS, as reflected by the absence of aggregate formation over the course of 7 days, suitable for biological applications (Figure 3i).

**FIGURE 3:**
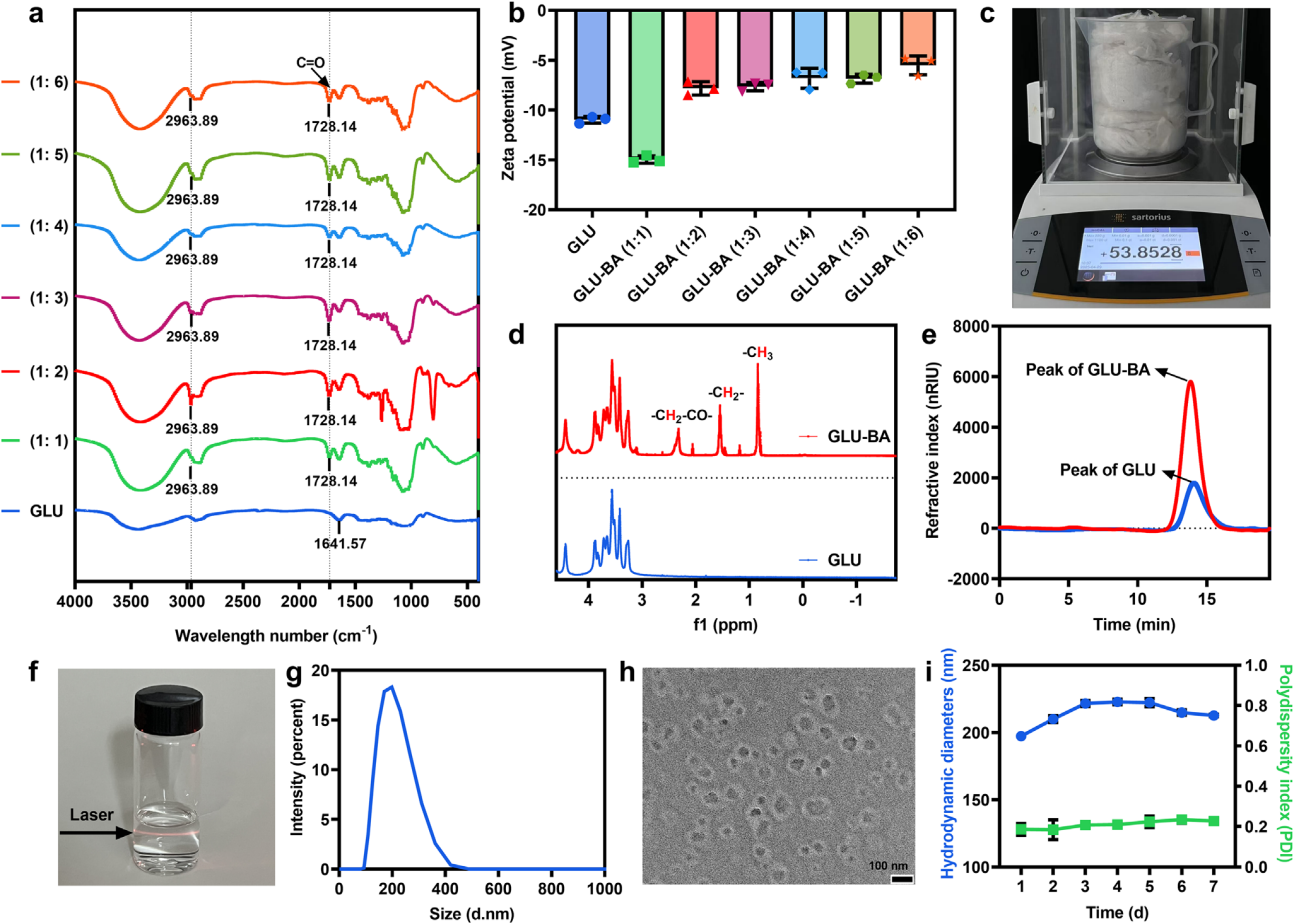
Characterization of GLU‐BA. (a) FTIR spectra of GLU and GLU‐BA synthesized at different GLU‐to‐BA molar ratios. (b) Zeta potential analysis of GLU and GLU‐BA synthesized at different GLU‐to‐BA molar ratios, *n* = 3 per group. (c) Photograph of GLU‐BA (∼54 g) synthesized via a one‐pot reaction. (d) ^1^H NMR spectra of GLU and GLU‐BA. (e) HPGPC analysis of GLU and GLU‐BA. (f) Photograph of GLU‐BA aqueous solution. (g) Hydrodynamic diameter of GLU‐BA in an aqueous environment, as measured by DLS. (h) TEM image of GLU‐BA in an aqueous environment. (i) Stability of GLU‐BA in PBS over 7 days, *n* = 3 per group.

### 2.3 Sequential Production of BA in the Intestine With Burst yet Gradual Dynamics Through Oral Administration of GLU‐BA

We then evaluated the BA production behavior of GLU‐BA after oral administration. Simulated gastrointestinal digestion was first performed on GLU‐BA. As shown in Figure 4a, negligible BA production from GLU‐BA was observed over 4 h (the period of emptying substances from the stomach after oral consumption is usually less than 2 h [29]) in simulated gastric fluid (SGF, pH = 2.4; see Experimental Section for the detailed formula), indicating its stability in the upper digestive tract. In striking contrast, when co‐incubated with simulated intestinal fluid (SIF, pH = 7.0; see Experimental Section for the detailed formula), a burst BA release from GLU‐BA within the initial 5 min was noticed. TEM images intuitively evidenced the structural collapse of GLU‐BA nanomicelles in SIF (Figure 4b). Continuous DLS monitoring of GLU‐BA nanomicelles incubated in SGF or SIF further indicated that GLU‐BA nanomicelles remained structurally stable in SGF but underwent swelling followed by structural transformation and gradual disassembly in SIF, suggesting an intestine‐responsive behavior (Figure S11). Collectively, these results provide strong evidence suggesting that GLU‐BA nanomicelles maintain structural integrity during gastric transit while undergoing intestine‐triggered disassembly, which should be attributed to the hydrolysis of ester bonds in GLU‐BA by high concentrations of the esterase pancreatin within the intestine [30]. Moreover, we speculated that the GLU simultaneously exposed during the disassembly of GLU‐BA nanomicelles would be subsequently metabolized by gut microbiota due to the prebiotic property of GLU [21], thereby powering intestine‐targeted BA production. To verify this, an in vitro fermentation assay was conducted by incubating GLU or GLU‐BA with fecal microorganisms. As fermentation proceeded, the GLU content gradually decreased (Figure 4c; Figure S12), accompanied by a gradual production of BA (Figure 4d; Figure S13) and simultaneous growth of bacteria (Figure 4e; Figure S14) in both cases of GLU and GLU‐BA, highlighting the rationality of choosing GLU as the substrate for constructing the oral formulation.

**FIGURE 4:**
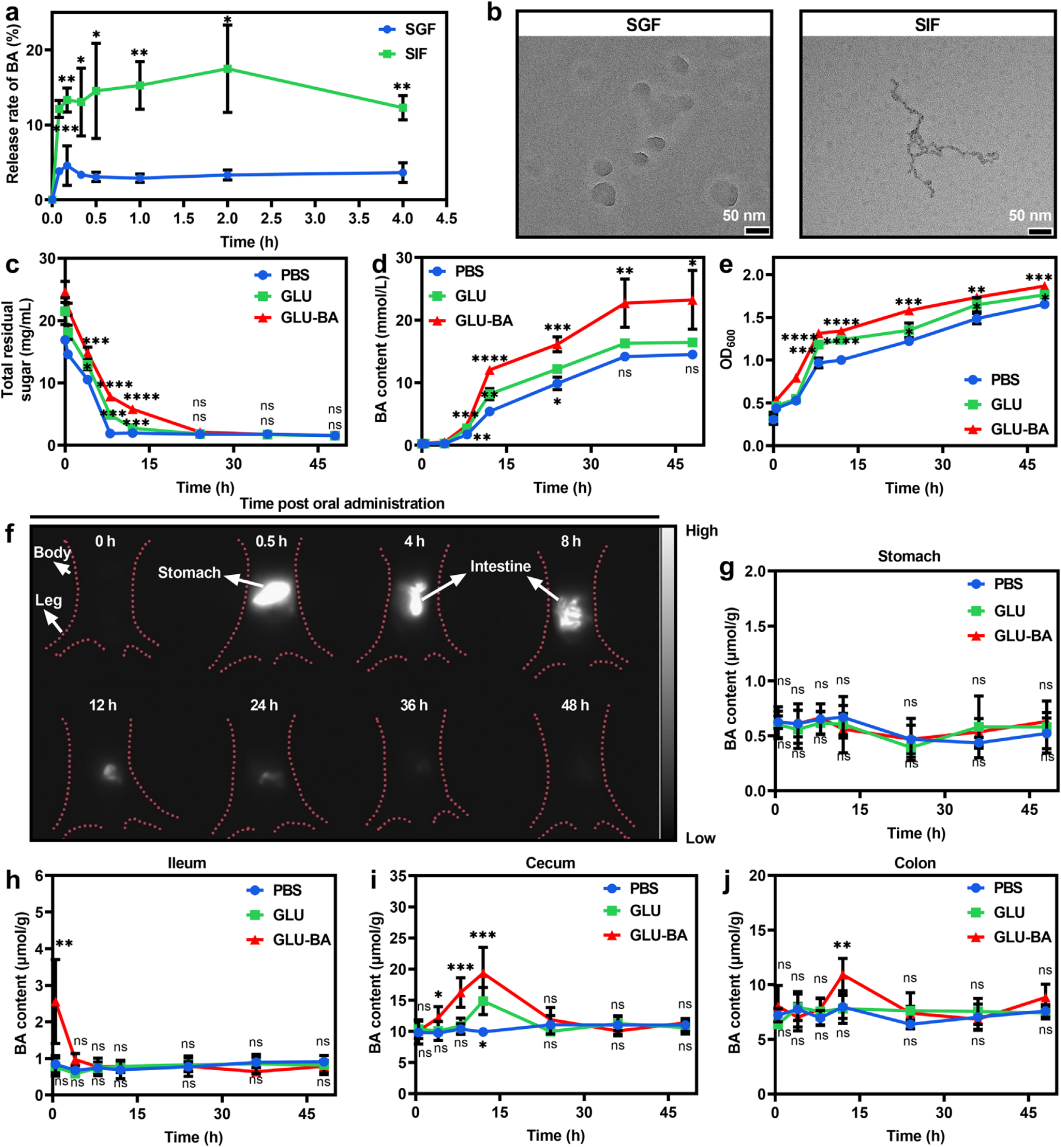
Evaluation of the intestinal responsiveness of GLU‐BA. (a) In vitro BA release dynamics of GLU‐BA in SGF or SIF, *n* = 3 per group. (b) TEM images of GLU‐BA in SGF or SIF. (c, d) Variation of total residual sugar amounts (c) and BA contents (d) over time in intestinal microbial fermentation assays, *n* = 3 per group. (e) Bacterial growth curves during in vitro fermentation. Bacterial growth was determined by measuring absorbance at 600 nm (i.e., optical density [OD_600_]), *n* = 3 per group. (f) Second near‐infrared (NIR‐II) fluorescence imaging (long‐pass filter: 1100 nm; laser: 808 nm; exposure time: 10 ms) of normal mice administered ICG‐GLU‐BA at different post‐administration time points. (g‐j) In vivo BA production dynamics in the stomach (g), ileum (h), cecum (i), or colon (j) after oral administration of PBS, GLU, or GLU‐BA to mice, *n* = 5 per group. Statistical analyses were performed using Student's t‐test and one‐way ANOVA. ns represents no significance compared with the PBS group. *< 0.05, **< 0.01, ***< 0.001, and ****< 0.0001 represent different levels of statistical significance.

Supported by the in vitro results, we then proceeded to profile the in vivo replenishment of BA after oral administration of GLU‐BA, for which the gastrointestinal biodistribution of GLU‐BA was first investigated. To achieve this, GLU‐BA was labelled with an indocyanine green fluorophore (ICG‐GLU‐BA; Figure S15; see Experimental Section for the detailed labeling process) and then orally administered to mice, followed by real‐time fluorescence monitoring. It was clearly observed that ICG‐GLU‐BA reached the intestine at 4 h post administration and was gradually excreted from the body due to intestinal peristalsis, with no signals detected at 48 h post administration (Figure 4f). Guided by the imaging results, we determined BA contents in the gastrointestinal tracts of mice at 0.5, 4, 8, 12, 24, 36, and 48 h after a single oral administration of GLU‐BA, including mice that received GLU (equivalent to the GLU content in GLU‐BA) or PBS for comparison. The results showed that at all detection time points, BA contents in the stomachs of PBS‐, GLU‐, or GLU‐BA‐treated mice were negligible (Figure 4g). Notably, as early as 0.5 h post administration, significant replenishment of BA was observed in the intestines (i.e., ileum; Figure 4h) of GLU‐BA‐treated mice, lasting for at least 12 h (i.e., cecum and colon; Figure 4i,j). In contrast, noticeable BA replenishment in the intestines of GLU‐treated mice did not occur until 8 h post administration. The highest amount of BA was detected in the cecum of GLU‐BA‐treated mice at 12 h post administration, reaching approximately two times that of PBS‐treated mice (Figure 4i). Combining all findings, it was concluded that, unlike the direct absorption in the stomach observed with NaBA administration (Figure 2l–o), GLU‐BA nanomicelles can traverse the stomach in an intact state. Subsequently, the intestinal environment rapidly induced the breakage of built‐in ester bonds in GLU‐BA for the burst release of BA and exposure of GLU, with the latter further metabolized by the gut microbiota residing within the cecum [31] and colon [32] to gradually produce BA in situ. Such a sequential BA intestinal production mode, enabled by oral GLU‐BA administration, achieved burst yet gradual replenishment kinetics, hopefully offering both prompt intervention and sustained therapeutic efficacy in BA‐mediated treatments.

### 2.4 Systematic Evaluation of Biocompatibility of GLU‐BA

Considering that the substrates used to construct GLU‐BA were the prebiotic and host‐beneficial BA moiety, GLU‐BA should demonstrate excellent biocompatibility, a prerequisite for in vivo applications. On this basis, a systematic biocompatibility evaluation of GLU‐BA was performed, including assessment of acute, sub‐chronic, and long‐term toxicity.

Acute biotoxicity study was performed according to the instruction of the Organization for Economic Cooperation and Development (OECD) Test Guidelines 425 (the guidance for assessing components’ impacts on human health and wildlife) [33]. Specifically, mice were randomly divided into two groups and were orally administered 200 µL PBS (denoted as PBS group) and GLU‐BA with the amount of 850 mg/kg per mouse (denoted as GLU‐BA group), respectively, wherein the administered GLU content exceeded by over 100‐fold the maximum recommended human intake (i.e., 100–500 mg per person) [20]. All the mice were continuously observed for 14 days and then sacrificed for further analysis (Figure 5a). During the observation period, no sudden death, abnormal behaviors, and differences in the body weight variation (*p* = 0.1994; Figure 5b) or in food/water intake (Figure S16) were observed among the mice, suggesting that the extremely high concentration of GLU‐BA did not affect normal physiological activities. Additionally, no significant differences were detected in major organ weights (Figure 5c–g), liver function indices (Figure S17), kidney function indices (Figure S18), and cardiac enzyme profile (Figure S19) between these two groups. Eventually, as revealed by H&E staining of major organs harvested from all the mice (Figure 5h), it was clear that the administration of extremely high concentration of GLU‐BA did not cause pathological variations, strongly confirming the negligible biotoxicity of GLU‐BA.

**FIGURE 5:**
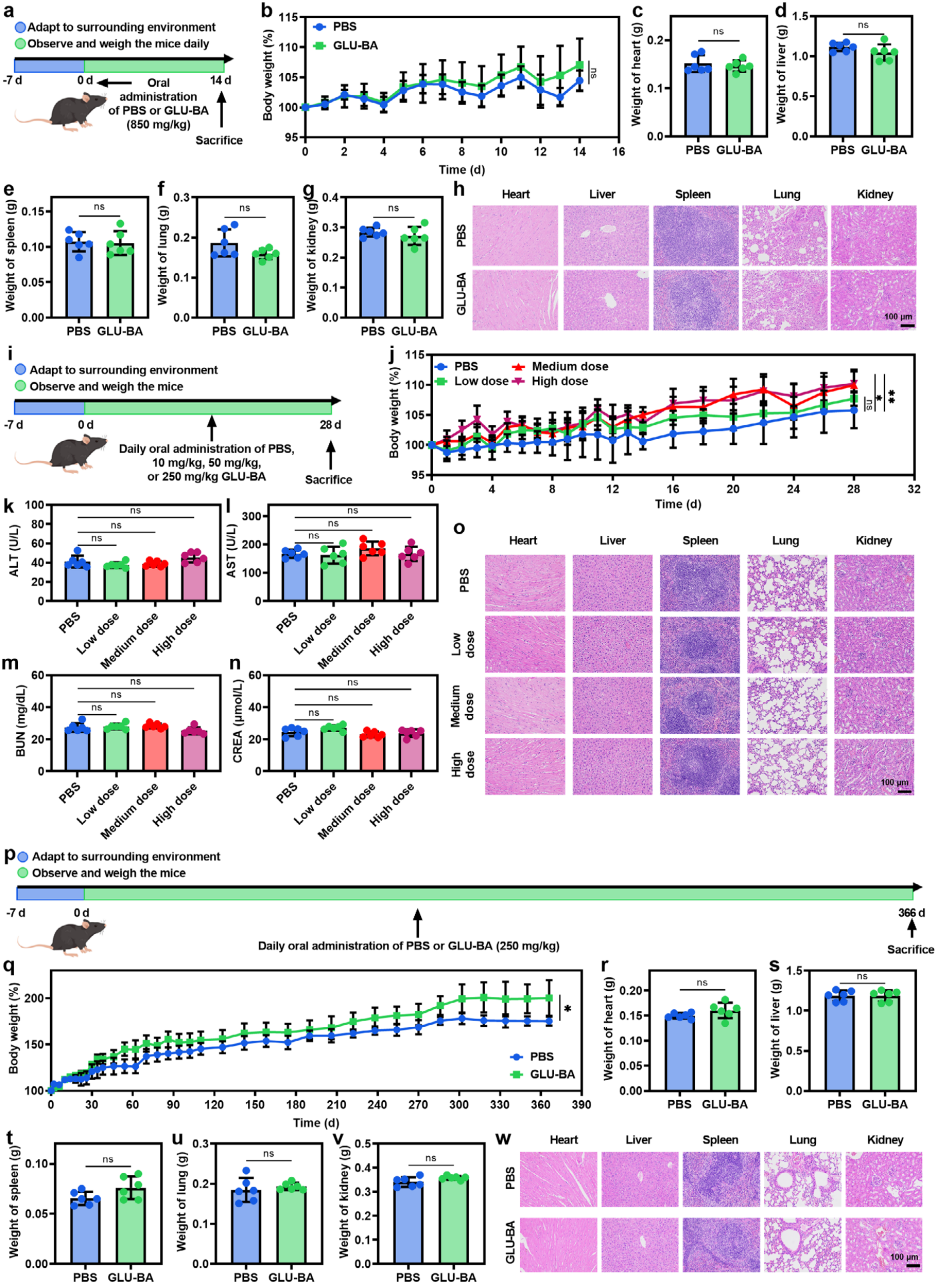
Evaluation of acute toxicity, sub‐chronic toxicity, and chronic toxicity of GLU‐BA in normal mice. (a) Schematic illustrating the acute toxicity evaluation. C57BL/6J mice were orally administered PBS or GLU‐BA (850 mg/kg) on the first day, and then observed and weighed for 14 consecutive days. All the mice were sacrificed on day 14 to collect organs and blood for further analysis. (b) Changes in the body weight over time after oral administration of PBS or GLU‐BA, *n* = 6 per group. (c‐g) Organ weights of mice from the PBS and GLU‐BA groups, including heart (c), liver (d), spleen (e), lung (f), and kidney (g), *n* = 6 per group. (h) H&E staining of major organs harvested from mice in the PBS and GLU‐BA groups. (i) Schematic illustrating the sub‐chronic toxicity evaluation. C57BL/6J mice were orally administered PBS, GLU‐BA (10 mg/kg; low dose), GLU‐BA (50 mg/kg; medium dose), or GLU‐BA (250 mg/kg; high dose) on a daily basis, and then observed and weighed for 28 consecutive days. All the mice were sacrificed on day 28 to collect organs and blood for further analysis. (j) Changes in the body weight over time after oral administration of PBS, low‐dose, medium‐dose, and high‐dose GLU‐BA, respectively, *n* = 6 per group. (k, l) Liver function analysis of mice from the PBS, low‐dose, medium‐dose, and high‐dose GLU‐BA groups, including alanine aminotransferase (ALT; k) and aspartate transaminase (AST; l), *n* = 6 per group. (m, n) Kidney function analysis of mice from the PBS, low‐dose, medium‐dose, and high‐dose GLU‐BA groups, including blood urea nitrogen (BUN; m) and creatinine (CREA; n), *n* = 6 per group. (o) H&E staining of major organs harvested from mice in the PBS, low‐dose, medium‐dose, and high‐dose GLU‐BA groups. (p) Schematic illustrating the long‐term toxicity evaluation. C57BL/6J mice were orally administered PBS or GLU‐BA (250 mg/kg) on a daily basis, and then observed and weighed for 366 days. All mice were sacrificed on day 366 to collect organs and blood for further analysis. (q) Changes in the body weight over time after oral administration of PBS or GLU‐BA, *n* = 6 per group. (r‐v) Organ weights of mice from the PBS and GLU‐BA groups, including heart (r), liver (s), spleen (t), lung (u), and kidney (v), *n* = 6 per group. (w) H&E staining of major organs harvested from mice in the PBS and GLU‐BA groups. Statistical analyses were performed using Student's t‐test and one‐way ANOVA. ns represents no significance. *< 0.05 and **< 0.01 represent different statistical significances.

Subsequently, a sub‐chronic toxicity study was conducted according to the OECD Test Guidelines 407 [34]. Mice were randomized into four groups and received daily oral administration of PBS (denoted as PBS group) or GLU‐BA at doses of 10, 50, or 250 mg/kg (denoted as low‐, medium‐, and high‐dose GLU‐BA groups, respectively) for 28 consecutive days (Figure 5i). Clinical observations showed that the body weight profiles of mice in the low‐dose group were not significantly different from those in the PBS group (*p* = 0.3523), whereas with increasing doses, the body weights of mice in the medium‐dose (*p* = 0.0129) and high‐dose (*p* = 0.0088) groups were significantly higher than those in the PBS group (Figure 5j). Food and water intake situations were similar to the body weight profiles (Figure S20). Evaluation of organ weights among all the mice did not show significant differences (Figure S21), which was similar to the results of the blood biochemical index investigation (Figure 5k–n). H&E staining further verified that the continuous administration of different concentrations of GLU‐BA to mice caused negligible damage to the major organs (Figure 5o).

In most cases, the introduction of exogenous substances into the body inevitably results in long‐term toxicity, severely hindering the clinical translation of most therapeutic agents. Therefore, we were pushed forward to evaluate the long‐term biotoxicity of GLU‐BA (Figure 5p). During daily administration of GLU‐BA (250 mg/kg) for 366 consecutive days, no death and abnormal behaviors were observed among the mice. The body weights of mice in the GLU‐BA group were significantly higher than those in the PBS group in the late stage (*p* = 0.0114; Figure 5q), and adverse effects of long‐term administration of GLU‐BA on organ weights (Figure 5r–v), serum biochemical indices (Figure S22), and histopathological alterations in the major organs (Figure 5w) and colon (Figure S23) were negligible. Collectively, these results provided strong evidence that GLU‐BA exhibited excellent biocompatibility.

### 2.5 Alleviating Effects of GLU‐BA on DSS‐Induced UC in MICE

With the intestine‐targeted burst‐yet‐gradual production profile of BA and superior biocompatibility fully characterized, we were motivated to explore GLU‐BA as a therapeutic agent to deal with diseases associated with intestinal dysfunction, for which the DSS‐induced UC model was first chosen for demonstration. Before the systematic evaluation, we investigated the effect of the administered dosage on therapeutic efficacy. Specifically, C57BL/6 mice were administered 3% DSS in drinking water for 7 consecutive days to establish UC [35], followed by daily oral administration of low‐dose GLU‐BA (100 mg/kg), medium‐dose GLU‐BA (150 mg/kg), high‐dose GLU‐BA (200 mg/kg), or PBS (the model group) for another 6 consecutive days (Figure S24a). Compared to the PBS treatment, both the medium‐ and high‐dose GLU‐BA treatments significantly ameliorated the symptoms of weight loss (*p* = 0.0002; Figure S24b), DAI score elevation (*p* < 0.05; Figure S24c), colon length shortening (*p* < 0.01; Figure S25), and spleen index increase (*p* < 0.01; Figure S26). According to further in‐depth characterization, either medium‐ or high‐dose GLU‐BA treatment efficiently repaired colonic tissue damage (Figure S27), revealing a favorable therapeutic outcome. In contrast, none of the above improvements were observed with low‐dose GLU‐BA treatment. Therefore, medium‐dose GLU‐BA was selected for subsequent experiments.

To further verify the superiority of GLU‐BA in treating UC, we compared the therapeutic efficacy conferred by GLU (the applied dosage was equivalent to the GLU amount in GLU‐BA), 5‐aminosalicylic acid (5‐ASA; the first‐line drug for treating UC but with serious side effects [36]), or GLU‐BA. Normal mice administered PBS and UC mice administered PBS throughout the experimental period were designated as the normal and model groups, respectively (Figure 6a). As shown in Figure 6b, GLU‐BA‐treated UC mice rapidly regained their weights, which was consistent with the above results (Figure S24b), approaching that of mice treated with 5‐ASA. Notably, the intervention by GLU also prevented UC mice from persistently losing weights (*p* = 0.0145), possibly due to its prebiotic property [37] that can be metabolized by gut microbiota into BA, as verified above (Figure 4d,i,j). However, compared to the body weight recovery observed in the GLU‐BA group, GLU treatment could only prevent progression of weight loss. These findings should be attributed to the initial burst release of BA resulting from the rapid hydrolysis of ester bonds in GLU‐BA, as identified above (Figure 4a,h), thus initiating a prompt intervention. Such a process could facilitate the therapeutic action to fall within the early treatment window, a critical period for initiating recovery. This stands in sharp contrast to approaches relying solely on the gradual supplementation of therapeutic components, which were limited to preventing disease progression rather than actively promoting recovery (see “Gradual supplementation” column in Table S3). Consistently, the evaluation of DAI scores (Figure 6c) indicated that the best therapeutic performance was achieved by GLU‐BA or 5‐ASA treatment, which was intuitively supported by the colon length (Figure 6d,e) and spleen indices (Figure 6h). Compared to the model and GLU groups, GLU‐BA treatment restored the damage to the colonic epithelium to a normal state in UC mice, featuring an intact and regular finger‐like crypt structure, as well as the existence of abundant goblet cells (Figure 6f,g). Based on these findings, we then investigated the expression of tight junction proteins, of which ZO‐1 and Occludin play crucial roles in the maintenance of mechanical integrity and normal function of the intestinal barrier [38, 39], in the colonic tissues of mice across different groups via immunofluorescence staining. As noted by results, GLU‐BA markedly increased the expression levels of ZO‐1 (Figure 6i,j) and Occludin (Figure 6k,l), surpassing the GLU treatment and approaching the normal/5‐ASA groups. As anticipated, the GLU‐BA group exhibited a significant decrease in the secretion of pro‐inflammatory cytokines (i.e., tumor necrosis factor‐α [TNF‐α], Figure 6m; interleukin‐6 [IL‐6], Figure 6n) and an increase in the expression levels of immunoregulatory cytokines (i.e., transforming growth factor‐β [TGF‐β], Figure 6o; interleukin‐10 [IL‐10], Figure 6p) compared to the model group. Importantly, in contrast to other studies based solely on the burst release of therapeutic components for UC treatment, which typically administered at dosages of 200–1200 mg/kg per day for durations of 7–10 days (see “Burst release” column in Table S3), our strategy required only 150 mg/kg per day to achieve recovery within only 6 days. This effect should be ascribed to the in situ gradual production of BA by GLU‐BA, as confirmed above (Figure 4i,j), to compensate for its rapid intestinal metabolism, thereby maintaining a considerable BA level in the intestine for an extended period (at least 12 h) to greatly improve and prolong its pharmaceutical action.

**FIGURE 6:**
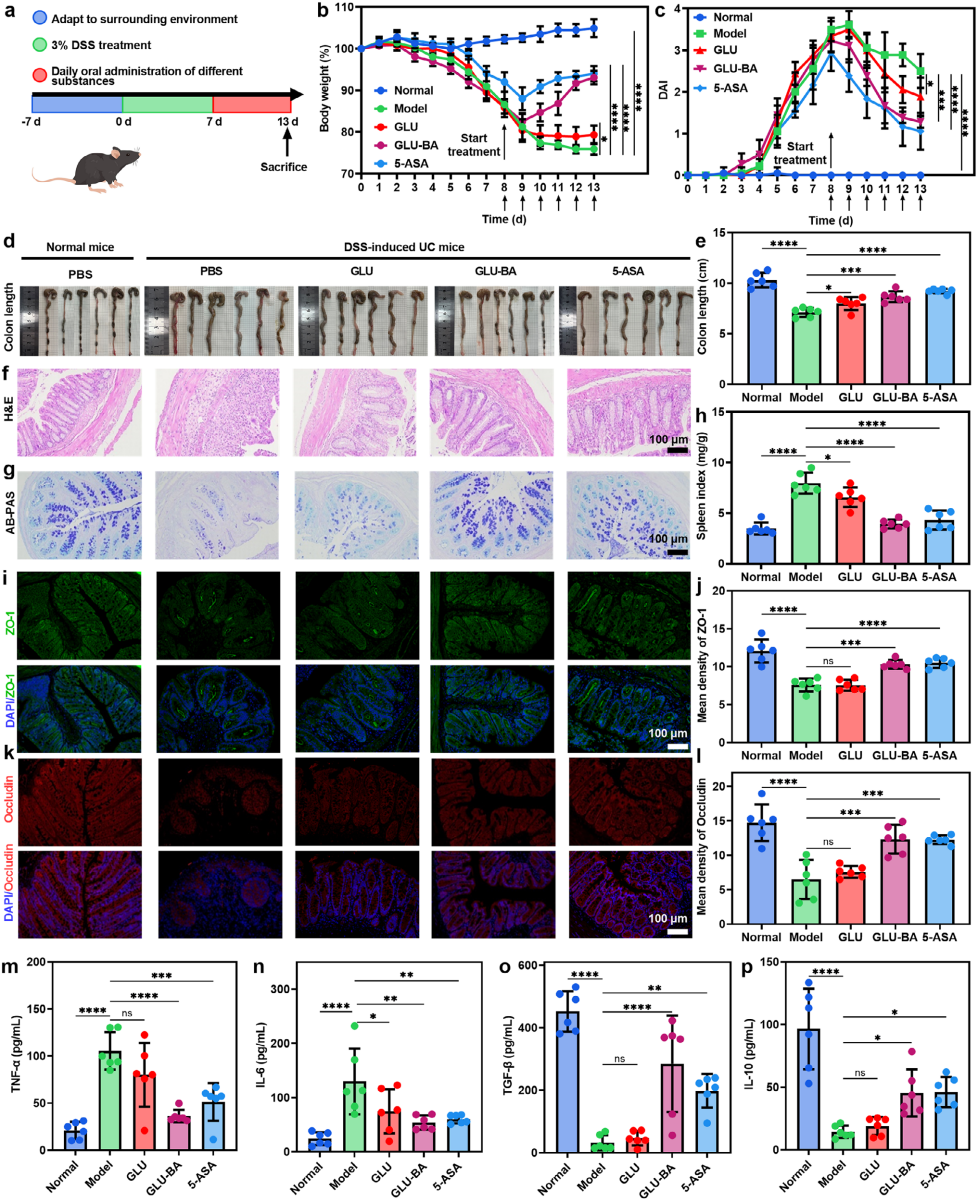
Oral administration of GLU‐BA reversed the progression of DSS‐induced UC in mice. (a) Schematic illustrating the intervention of UC in mice. C57BL/6J mice were given drinking water containing 3% DSS for 7 continuous days to establish a mouse UC model. Afterward, PBS, GLU, GLU‐BA, or 5‐ASA was given for 6 consecutive days by daily oral administration. (b) Changes in the body weight of mice over time among different groups, *n* = 6 per group. (c) Changes in DAI of mice over time among different groups, *n* = 6 per group. (d) Photographs of colons of mice from different groups, *n* = 6 per group. (e) Corresponding quantified lengths of the colons of mice from different groups, *n* = 6 per group. (f) Representative H&E staining images of colon tissues harvested from mice across different groups. (g) Representative Alcian blue‐Periodic acid‐Schiff (AB‐PAS) staining images of colon tissues harvested from mice across different groups. (h) Spleen indices of mice from different groups, *n* = 6 per group. (i, j) Representative immunofluorescence staining images showing the expression of tight junction protein ZO‐1 (i) and the corresponding quantitative analysis (j). The nucleus was stained with DAPI. (k, l) Representative immunofluorescence staining images showing the expression of tight junction protein Occludin (k) and the corresponding quantitative analysis (l). (m‐p) Secretion levels of inflammation‐related cytokines in mice from different groups, *n* = 6 per group, including TNF‐α (m), IL‐6 (n), TGF‐β (o), and IL‐10 (p). Statistical analyses were performed using one‐way ANOVA. ns represents no significance. *< 0.05, **< 0.01, ***< 0.001, and ****< 0.0001 represent different statistical significances.

Moreover, compared with the physical mixture of GLU and BA (GLU+BA) containing equivalent amounts of GLU and BA, GLU‐BA exhibited enhanced therapeutic efficacy in DSS‐induced acute UC model, further highlighting the rationale of our design (Figure S28). These findings demonstrate that the therapeutic benefits of GLU‐BA cannot be attributed merely to the co‐administration of GLU and BA, but instead arise from the engineered delivery strategy, which enhances BA availability at the intestinal site. To further evaluate whether GLU‐BA could provide sustained protection under prolonged inflammatory conditions, a DSS‐induced chronic UC model was established. Compared with the model group, GLU‐BA administration promoted body weight recovery and attenuated colonic injury (Figure S29). Histological examination further demonstrated that GLU‐BA treatment alleviated intestinal barrier disruption, as evidenced by reduced tissue damage in H&E staining and enhanced mucus production in AB‐PAS staining. The sustained efficacy of GLU‐BA under chronic inflammatory conditions further supports the advantages of this engineered BA supplementation strategy and highlights its potential as a promising platform for targeted BA supplementation. In summary, oral administration of GLU‐BA enabled the sequential production of BA to achieve the burst yet gradual replenishment in the intestine, balancing prompt intervention and sustained therapeutic efficacy for effective management of UC.

### 2.6 Biological Responses and Mechanistic Insights Underlying Intestinal Protection

Encouraged by the above satisfactory therapeutic outcomes, we then began to decipher the underlying biological responses and mechanistic insights. As shown in Figure 7a, the BA contents in the mouse feces of the GLU‐BA group were significantly higher than those in both the model (*p* < 0.0001) and GLU (*p* = 0.0287) groups. It was reported that BA could exert anti‐inflammatory functions by binding G protein‐coupled receptor 109A (GPR109A), promoting the differentiation of T‐regulatory immune cells to up‐regulate IL‐10 expression [40]. Therefore, we employed GPR109A blockade as a functional validation approach to determine whether BA produced from GLU‐BA contributes to its therapeutic effects. To exclude the possibility that mepenzolate bromide (MB), a commercial GPR109A inhibitor [41], itself affected UC progression, a MB‐only group was first included. Compared with the DSS‐induced UC model group, MB administration alone did not significantly alter disease severity (Figure S30). Based on this validation, we subsequently investigated whether pharmacological blockade of GPR109A would affect the therapeutic efficacy of GLU‐BA. UC mice were treated with MB to inhibit BA–GPR109A signaling, followed by oral administration of GLU‐BA (Figure 7b). Notably, the therapeutic efficacy of GLU‐BA against UC was attenuated once MB was introduced, as indicated by the shorter colon length observed in MB/GLU‐BA‐treated mice (*p* = 0.0212; Figure 7c,d). In addition, H&E staining showed that in contrast to the normal state of colons from GLU‐BA‐treated mice, those from MB/GLU‐BA‐treated mice exhibited damaged structures characterized by branching, atrophic, and irregular intestinal crypts (Figure 7e). Moreover, the introduction of MB prevented GLU‐BA treatment from restoring the expression of tight junction proteins ZO‐1 and Occludin (Figure 7f–i). Collectively, these results strongly corroborated the therapeutic role of BA in UC.

**FIGURE 7:**
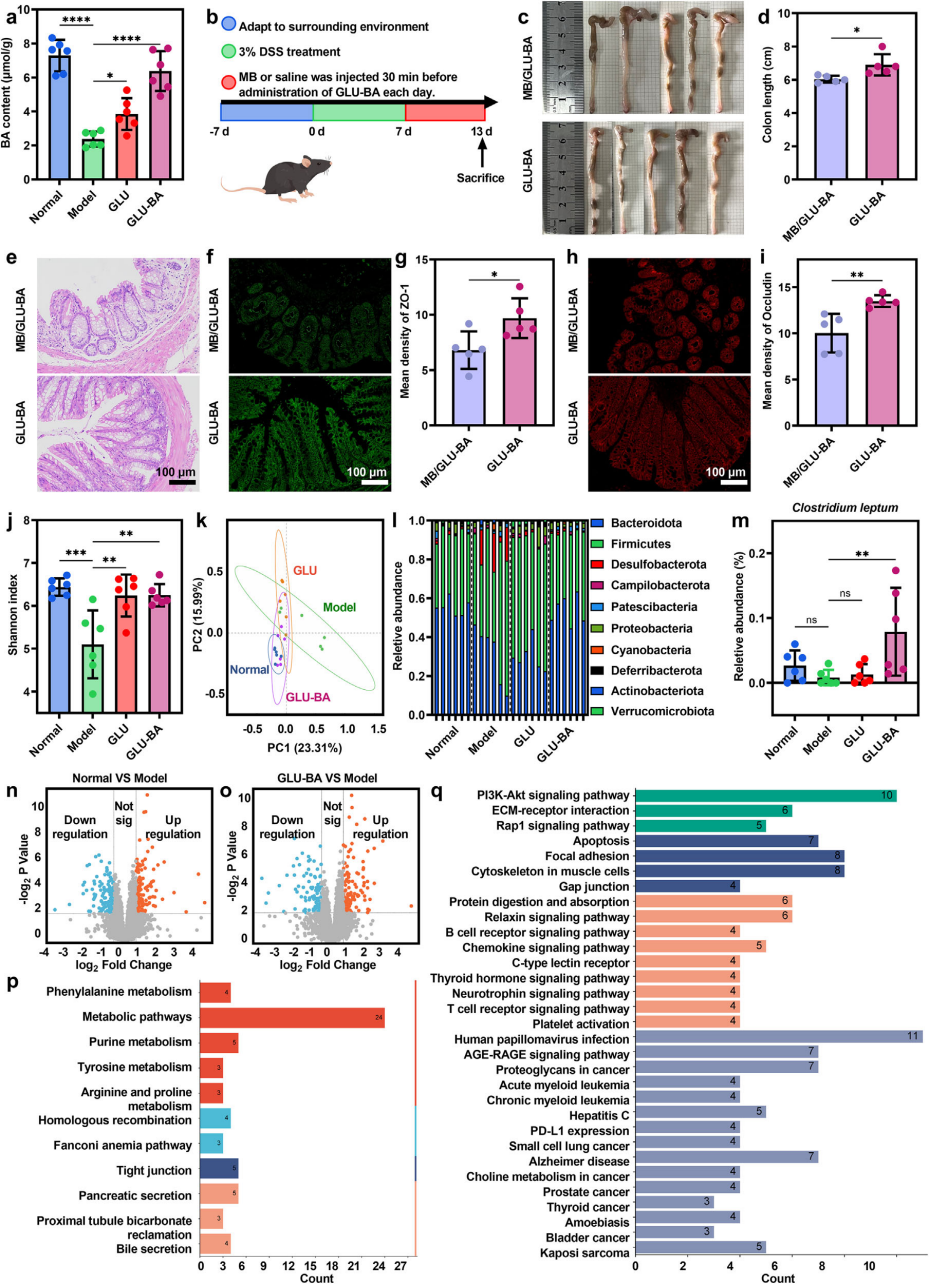
Mechanistic insights into the alleviation of DSS‐induced UC. (a) Analysis of BA contents in the feces of mice from different groups, *n* = 6 per group. (b) Schematic illustrating the experiment regarding the inhibition of BA function in vivo. C57BL/6J mice were given drinking water containing 3% DSS for 7 continuous days to establish a mouse UC model. Afterward, MB or saline was administered intraperitoneally. 30 min later, GLU‐BA was orally administered. Above processes were repeated daily for 6 continuous days. (c) Representative colon photographs of mice from different groups at the end of the intervention, *n* = 5 per group. (d) Corresponding quantified colonic lengths of mice from different groups, *n* = 5 per group. (e) Representative H&E staining images of colon tissues of mice from different groups. (f, g) Representative immunofluorescence staining images showing the expression of tight junction protein ZO‐1 (f) and the corresponding quantitative analysis (g). (h, i) Representative immunofluorescence staining images showing the expression of tight junction protein Occludin (h) and the corresponding quantitative analysis (i). (j) α‐diversity of the gut microbiota across different groups according to the Shannon index analysis, *n* = 6 per group. (k) β‐diversity analysis determined by PCoA based on the Bray‐Curtis distance of mice from different groups, *n* = 6 per group. (l) Top 10 most abundant gut microbiota at the phylum level across different groups, *n* = 6 per group. (m) Relative abundance of *Clostridium leptum* (%) in mice from different groups, *n* = 6 per group. (n) Volcano plot illustrating differentially expressed proteins in colonic tissues between the normal and model groups (*p* < 0.05, |log_2_(Fold Change)| > log_2_(1.5)), *n* = 6 per group. “Not sig” represents no significance. (o) Volcano plot illustrating differentially expressed proteins in colonic tissues between the GLU‐BA and model groups (*p* < 0.05, |log_2_(Fold Change)| > log_2_(1.5)), *n* = 6 per group. “Not sig” represents no significance. (p) KEGG enrichment analysis of up‐regulated proteins in the GLU‐BA group compared to the model group, *n* = 6 per group. (q) KEGG enrichment analysis of down‐regulated proteins in the GLU‐BA group compared to the model group, *n* = 6 per group. Statistical analyses were performed using Student's t‐test and one‐way ANOVA. ns represents no significance. *< 0.05, **< 0.01, ***< 0.001, and ****< 0.0001 represent different statistical significances.

Beyond BA's primary contribution, we speculated that GLU‐BA might also participate in the modulation of the gut microbiota to reinforce therapeutic efficacy, as suggested by the in vitro fermentation assay (Figure 4e) and considering the prebiotic property of the GLU substrate [20]. To verify this hypothesis, we analyzed the composition and abundance of fecal microorganisms to evaluate the effect of GLU‐BA on gut microbiota regulation in UC mice. The Shannon index curve gradually flattened as the number of samples increased, indicating that the sequencing depth was adequate for further analysis of species richness (Figure S31). Compared to the model group, GLU or GLU‐BA administration significantly enhanced the α‐diversity of the gut microbiota, as reflected by the Shannon index (*p* < 0.01; Figure 7j) in UC mice. According to PCA (Figure S32) and principal coordinate analysis (PCoA; Figure 7k) based on the Bray‐Curtis distance, GLU‐BA treatment reshaped the β‐diversity profile of the gut microbiota in UC mice toward the one similar to that of normal mice. Worth mentioning, GLU alone also improved the β‐diversity to some extent, suggesting its prebiotic role and further illustrating the rationality of our design. Next, we conducted a detailed analysis of the composition of the gut microbiota across different groups at the phylum level. The results showed that only the profile of the gut microbiota in the GLU‐BA group was comparable to that in the normal group (Figure 7l), indicating the rebalance of intestinal microbial communities. Further analysis at the phylum level revealed that treatment with GLU‐BA significantly decreased the relative abundance of *Proteobacteria* (*p* = 0.0117; Figure S33) in UC mice, which include toxin‐secreting bacteria that interfere with host cellular processes [26]. Additionally, GLU‐BA treatment increased the abundance of *Bacteroidia* (*p* = 0.0018; Figure S34), a prominent category of beneficial bacteria known for their roles in polysaccharide degradation [42, 43], in UC mice. At the genus level, the abundance of *Escherichia‐Shigella*, a pathogenic member associated with exacerbated intestinal inflammation [44], was significantly enriched in the model group and such enrichment was efficiently mitigated by GLU‐BA administration (*p* = 0.0112; Figure S35). Notably, the relative abundance of *Clostridium leptum*, a group of bacteria known to produce BA [45], were significantly increased in the GLU‐BA group compared to the model group (*p* = 0.0099; Figure 7m), demonstrating to be an endogenous contributor to advance sustained BA replenishment in the intestine.

To gain deeper insights into the therapeutic effects of GLU‐BA, proteomic analysis was performed on colonic tissues. As shown by volcano plots, compared to the normal group, 121 proteins were significantly down‐regulated and 125 proteins were up‐regulated in the model group (Figure 7n), revealing an abnormal protein profile. Notably, after intervention by GLU‐BA, 139 proteins were significantly up‐regulated, and 92 proteins were significantly down‐regulated (Figure 7o). The Kyoto Encyclopedia of Genes and Genomes (KEGG) enrichment analysis revealed that the differentially expressed proteins were implicated in 42 signaling pathways (Figure 7p,q). Among them, the “purine metabolism” signaling pathway was significantly enriched after GLU‐BA intervention (Figure 7p), which is reported to be closely related to the regulation of the ecology of the intestinal flora [46]. In addition, the “tight junction” signaling pathway also appeared in the GLU‐BA group, reinforcing the above findings (Figure 6i–l). Moreover, the “PI3K‐Akt signaling pathway” and “ECM‐receptor interaction” terms, which are closely associated with the exacerbation of inflammatory responses, were significantly down‐regulated in the GLU‐BA group (Figure 7q), consistent with the above therapeutic outcomes (Figure 6m–p). Collectively, these findings deciphered that oral administration of GLU‐BA could ameliorate the dysbiosis of the gut microbiota, coordinating BA produced in situ to regulate the colonic protein profile toward a state conducive to restoring UC‐induced damage.

### 2.7 Versatility of GLU‐BA as an Oral Therapeutic Agent

Considering the broad‐spectrum bioactivities of BA in the gut, such as maintaining intestinal homeostasis through intestinal immunoregulation [47, 48, 49, 50], GLU‐BA demonstrates great potential as a versatile oral therapeutic. To confirm this, we established a Cy‐induced immunosuppressive model for evaluation. Specifically, mice were intraperitoneally injected with 80 mg/kg of Cy for 3 consecutive days to induce immunosuppression [51]. Based on this model, the therapeutic efficacy of GLU, GLU‐BA, and levamisole hydrochloride (LH, positive control group; an immune enhancer widely used in cancer treatment but often associated with serious side effects, such as dizziness and vomiting) was then compared in parallel. Normal mice orally administered PBS and immunosuppressive mice orally administered PBS throughout the experimental period were designated as the normal and model groups, respectively (Figure 8a). As shown in Figure 8b and Figure S36, immunosuppressive mice treated with GLU‐BA gradually regained the body weight and food/water intake, approaching levels observed in the LH‐treated group and eventually becoming similar to those in the normal group. Similar to the results observed in the above UC case, GLU only prevented the body weight from consistently losing, suggesting that the delayed BA production missed the early therapeutic window, thereby compromising GLU's ability to reverse disease progression. Similar trends were also observed for the spleen and thymus indices (Figure 8c,d), further supporting the rationality of GLU‐BA design. Notably, GLU‐BA treatment also repaired intestinal epithelial damage in immunosuppressive mice, restoring to a normal state with intact and regularly structured finger‐like crypts, as well as with abundant goblet cells (Figure 8e and Figure S37). Based on these findings, we then profiled immune homeostasis in the intestinal lamina propria across different groups. Revealed by Figure 8f,g, intraperitoneal injection of Cy resulted in a significant decrease of the number of CD4^+^ and CD8^+^ T cells, whereas GLU‐BA treatment effectively reversed this depletion back to a normal state, preliminarily verifying the immunostimulatory effects [52]. In addition, we found that GLU‐BA treatment could repair the compromised intestinal barrier in immunosuppressive mice, as evidenced by the restoration of the expression of tight junction proteins (i.e., ZO‐1 and Occludin; Figures S38 and S39), which are essential for maintaining intestinal immune balance. As a result, GLU‐BA treatment led to a significant reduction in the levels of TGF‐β (*p* = 0.0004; Figure 8h) and IL‐10 (*p* = 0.0242; Figure 8i), in comparison to the model group. Furthermore, GLU‐BA treatment markedly increased the secretion of immunoglobulin A (IgA; *p* = 0.0005; Figure 8j) and immunoglobulin G (IgG; *p* = 0.0193; Figure 8k) by 273.1% and 126.2%, respectively, relative to the model group, even surpassing the positive control (LH) group and approaching the normal group. Importantly, compared to previous studies based on oral formulations for addressing Cy‐induced immunosuppression, which typically required daily dosages of 200–1000 mg/kg over 10–30 days (Table S4), our strategy needed only 150 mg/kg per day to achieve recovery within just 7 days, further highlighting GLU‐BA's therapeutic superiority. Collectively, these findings confirmed that GLU‐BA possessed a robust capacity to restore immunodeficiency, demonstrating its therapeutic versatility.

**FIGURE 8:**
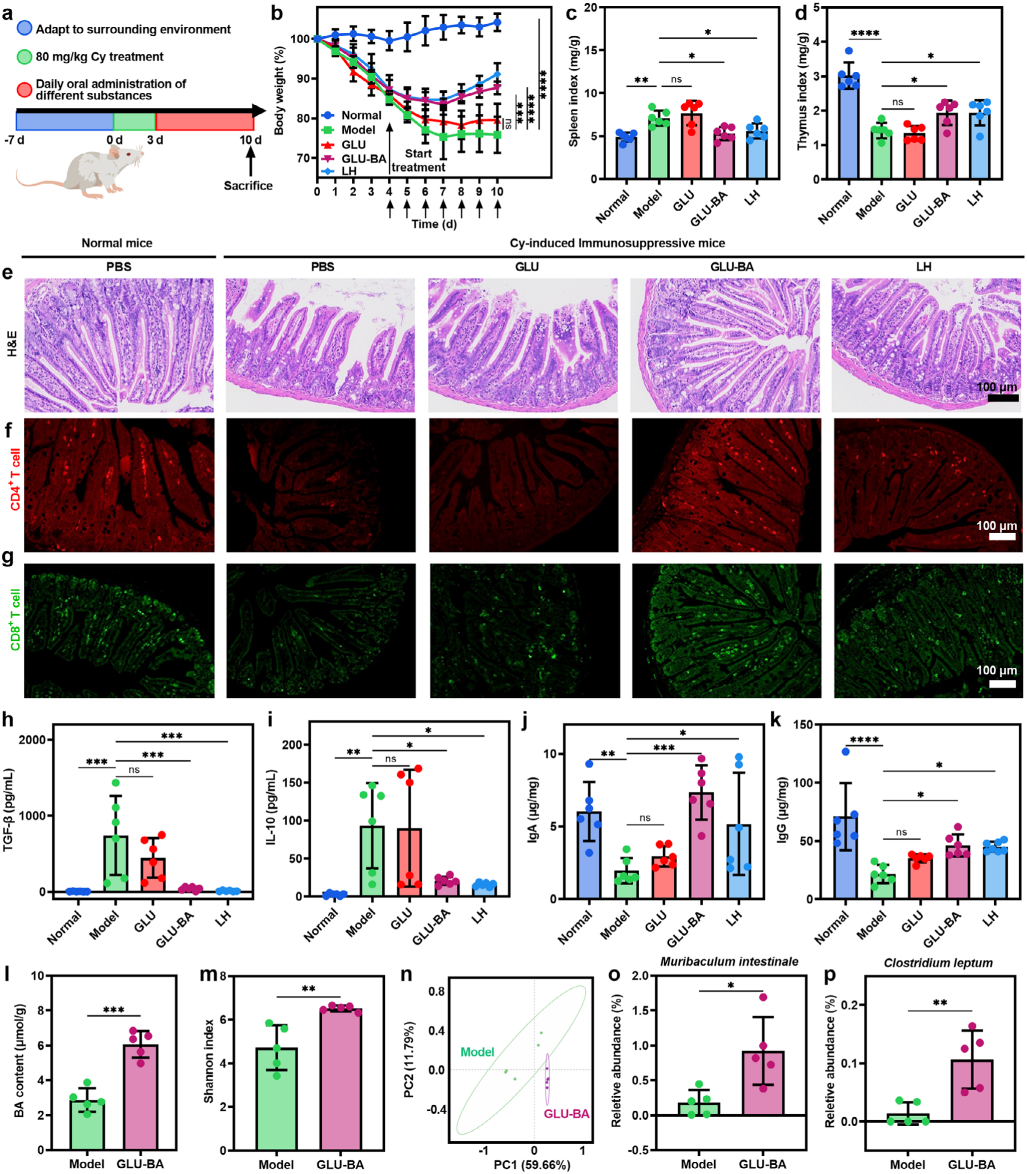
Therapeutic effects of GLU‐BA on Cy‐induced immunosuppressive mice. (a) Schematic illustrating the treatment process. BALB/c mice were given intraperitoneal injection of Cy for 3 continuous days to establish the immunosuppression model. Afterward, PBS, GLU, GLU‐BA, or LH was given for 7 continuous days by oral administration on a daily basis. (b) Changes in the body weight of mice over time among different groups, *n* = 6 per group. (c) Spleen indices of mice across different groups, *n* = 6 per group. (d) Thymus indices across different groups, *n* = 6 per group. (e) Representative H&E staining images of small intestinal tissues harvested from different groups. (f, g) Representative immunofluorescence staining images profiling immune cells in the intestines of mice from different groups, including CD4^+^ (f) and CD8^+^ T cells (g). (h, i) Secretion levels of TGF‐β (h) and IL‐10 (i) in small intestines of mice from different groups, *n* = 6 per group. (j, k) Secretion levels of typical immunoglobulins in small intestines of mice from different groups, including IgA (j) and IgG (k), *n* = 6 per group. (l) Analysis of BA contents in the feces of mice from the model and GLU‐BA groups, *n* = 5 per group. (m) α‐diversity of the gut microbiota across different groups according to the Shannon index analysis, *n* = 5 per group. (n) β‐diversity determined by PCoA based on the Bray‐Curtis distance of mice from the model and GLU‐BA groups, *n* = 5 per group. (o) Relative abundance (%) of *Muribaculum intestinale* in mice from the model and GLU‐BA groups, *n* = 5 per group. (p) Relative abundance (%) of *Clostridium leptum* in mice from the model and GLU‐BA groups, *n* = 5 per group. Statistical analyses were performed using Student's t‐test and one‐way ANOVA. ns represents no significance. * < 0.05, ** < 0.01, *** < 0.001, and **** < 0.0001 represent different statistical significances.

To elucidate the therapeutic mechanism of GLU‐BA for the above process, we first quantified BA levels in the feces collected from the GLU‐BA and model groups. Obviously, BA contents in the GLU‐BA group were significantly higher than those in the model group (Figure 8l; *p* = 0.0001), suggesting that BA plays a critical role in immune restoration, consistent with previous research [23]. Similar to the application of UC treatment, we also analyzed the composition and abundance of fecal microbiota to assess the regulatory effects of GLU‐BA on the gut microbiota in immunosuppressive mice. According to Figure S40, the sequencing depth was sufficient to further analyze species richness. Specifically, compared to the model group, oral administration of GLU‐BA significantly increased the α‐diversity (Figure 8m and Figure S41) and differentiated the β‐diversity (Figure 8n and Figure S42) of the gut microbiota in immunosuppressive mice, indicating the rebalance of microbial ecology. Furthermore, sequencing results revealed that GLU‐BA treatment reduced the relative abundance of *Proteobacteria* (*p* = 0.0493; Figure S43) and *Gammaproteobacteria* (*p* = 0.0430; Figure S44), which were reported to demonstrate pathogenicity [53], and increased the relative abundance of *Muribaculum intestinale* (SCFA‐producing bacteria [54]; *p* = 0.0125; Figure 8o) in immunosuppressive mice. In addition, bacteria with the ability to produce BA, that is, *Clostridium leptum* (Figure 8p; *p* = 0.0049), were also enriched in immunosuppressive mice after GLU‐BA intervention. Combing all the findings, it was concluded that GLU‐BA exerted the therapeutic versatility through intestinal BA production in combination with positive modulation of gut microbiota.

## 3 Conclusions

We developed a macromolecule (GLU‐BA) capable of sequentially producing BA in the intestine with burst yet gradual dynamics, achieving both prompt intervention and sustained therapeutic efficacy in treating DSS‐induced UC and Cy‐induced immunosuppression in mice. GLU‐BA was synthesized via a simple one‐step and easily‐scalable reaction, which covalently linked BA moieties to GLU through the ester bond selectively responsive to the intestinal environment, yielding approximately 54 g of inodorous GLU‐BA. Upon oral administration, GLU‐BA self‐assembled into nanomicelles in physiological conditions, preventing premature gastric absorption. Once reaching the intestine, endogenous esterases rapidly hydrolyzed ester bonds, triggering nanomicelles’ rupture for the burst release of BA, while simultaneously exposing GLU. The exposed GLU was subsequently metabolized by the gut microbiota, in situ producing BA gradually to maintain considerable intestinal BA levels for at least 12 h. As the substrates constructing GLU‐BA are the prebiotic and host‐beneficial BA moiety, it demonstrated excellent biocompatibility, with no observable toxicity in mice after 366 days of daily oral administration. With all advancements, once‐daily GLU‐BA administration at a dosage of 150 mg/kg significantly ameliorated DSS‐induced UC and Cy‐induced immunosuppression through the broad‐spectrum bioactivities of BA in the intestine. The sequential production of BA by GLU‐BA not only ensured prompt therapeutic intervention but also counteracted its rapid intestinal metabolism for sustained therapeutic efficacy, outperforming strategies based solely on burst or gradual supplementation. Additionally, oral administration of GLU‐BA restored gut microbiota homeostasis by enriching beneficial bacteria while simultaneously suppressing harmful bacteria to reinforce the therapeutic effectiveness. Of note, during such a rebalance, BA‐producing bacteria, such as *Clostridium leptum*, were also significantly enriched, demonstrating to be an endogenous source to further advance BA replenishment. By overcoming key limitations in intestinal BA supplementation, GLU‐BA offers a promising strategy for advancing disease management. Given its broad‐spectrum bioactivities, excellent biocompatibility, and scalable production, GLU‐BA holds the potential as a practical and safe approach for disease management, with the great potential for clinical translation or to be even adopted in daily health maintenance and disease prevention.

## 4 Experimental Section

### 4.1 Materials

GLU was obtained from Megazyme Co., Ltd. (Bray, Ireland). Butyric anhydride was purchased from Sigma‐Aldrich (St. Louis, MO, USA). ICG was sourced from Shaanxi Xinyan Bomei Biotechnology Co., Ltd. (Baoji, China). Enzyme‐linked immunosorbent assay kits for cytokines, including TNF‐α, IL‐6, TGF‐β, and IL‐10, were purchased from Boster Biological Technology Co., Ltd. (Wuhan, China). IgA and IgG were obtained from Shanghai MEIXUAN Science and Biological Technology Co., Ltd. (Shanghai, China). DSS with a molecular weight ranging from 36 000 to 50 000 Da was supplied by Alfa Aesar Co., Ltd. (Haverhill, USA). LH and Cy were obtained from Shanghai Aladdin Biochemical Technology Co., Ltd. (Shanghai, China). PBS and saline were purchased from SolarBio (Beijing, China).

### 4.2 Animals

Specific pathogen‐free (SPF) C57BL/6J mice (20 ± 0.2 g, male, aged 6–8 weeks) and BALB/c mice (20 ± 0.2 g, female, aged 6–8 weeks) were purchased from GemPharmatech Co., Ltd. (Nanjing, China). The mice were housed under controlled conditions: temperature of 23 ± 3°C, humidity of 55 ± 10%, a 12/12‐h light/dark cycle, and free access to food and water. Animal studies were conducted in accordance with the regulations approved by the Animal Care and Use Committee of Nanchang University (SYXK (Gan) 2021‐0004).

### 4.3 Metabolomics Analysis of UC Mice

C57BL/6J mice (20 ± 0.2 g, male, 6–8 weeks) were acclimated for 7 days, after which they were provided with drinking water containing 3% (w/v) DSS to establish the UC model, as described in our previous studies [26]. After the 7 consecutive days’ modeling period, 12 DSS‐induced UC mice were referred to as the model mice, while another 12 normal mice given drinking water were referred to as the normal mice. Fecal consistency, fecal bleeding, and body weight changes were monitored daily throughout the experiment. Each parameter was scored according to previously proposed criteria [55] and used to calculate the DAI. In the end, intestines were collected for detailed analysis.

The intestinal content samples stored at −80°C were thawed on ice. The thawed samples were homogenized using a grinder (30 Hz) for 20 s. After centrifugation at 3000 r/min for 30 s (4°C), a 400 µL solution (methanol: water = 7:3, v/v) containing the internal standard was added to 20 mg of the ground sample and shaken at 1500 rpm for 5 min. After incubation on ice for 15 min, the sample was centrifuged at 12 000 rpm for 10 min (4°C). A 300 µL aliquot of the supernatant was collected and stored at −20°C for 30 min. The sample was then centrifuged again at 12 000 rpm for 3 min (4°C), and a 200 µL aliquot of the supernatant was transferred for liquid chromatography‐mass spectrometry analysis.

### 4.4 Therapeutic Process of NaBA Toward DSS‐Induced UC in Mice

The model establishment was conducted as described in the section on *Metabolomics analysis of UC mice*. Starting on day 8, to evaluate the efficacy of NaBA in treating UC, 24 DSS‐induced UC mice were divided into two groups (*n* = 12) and administered NaBA (112 mg/kg, equivalent to the BA content in 200 mg/kg GLU‐BA) or PBS for 6 consecutive days. Normal mice administered PBS orally were designated as the normal group. Fecal consistency, fecal bleeding, and body weight changes were monitored daily throughout the experiment. Each parameter was scored according to previously proposed criteria [55] and used to calculate the DAI. At the end of the intervention, colonic tissues were collected for detailed analysis.

### 4.5 Synthesis of GLU‐BA

To synthesize GLU‐BA, 987 mg of GLU was first dissolved in dimethyl sulfoxide by heating it to 70°C, then transferred to a 500 mL three‐necked flask. After the system was cooled to room temperature, triethylamine and butyric anhydride (at molar ratios of 1:1, 1:2, 1:3, 1:4, 1:5, or 1:6) were added dropwise and allowed to react overnight. The GLU‐BA solid was obtained by freeze‐drying after removing small molecules via dialysis. For large‐scale synthesis of GLU‐BA, 45 g GLU was reacted with butyric anhydride at a molar ratio of 1:2, following the experimental procedure described above. DLS measurements of GLU‐BA in ultrapure water were performed using a Malvern Instruments Nano ZS instrument (ZS90, Malvern, UK). The chemical bonds in GLU‐BA were analyzed by FTIR, and the final molecular information was confirmed by NMR (^1^H). The relative molecular weights of GLU and GLU‐BA were determined using HPGPC (Agilent 1260, Agilent, USA).

### 4.6 In Vitro Digestive Simulation

We referred to the digestion protocol of INFOGEST 2.0 [56] and the previous in vitro digestive method adopted in the laboratory with appropriate modifications [57]. GLU‐BA was incubated in SGF (pH 2.4), containing 0.2% (w/v) sodium chloride and 3.2 mg/mL pepsin, with the pH adjusted using hydrochloric acid, for 4 h. Meanwhile, in a separate experiment, GLU‐BA was placed in SIF (pH 7.0), containing 0.68% (w/w) potassium dihydrogen phosphate, 10 mM bile salts, and 1% (w/w) pancreatin, with the pH adjusted using sodium hydroxide (NaOH), for 4 h. During incubation, the solutions were stirred at 180 rpm, and 1 mL aliquots were taken from the reaction mixture at specified time intervals for BA measurement.

### 4.7 In Vitro Fermentation Assay

Fresh feces from healthy male C57BL/6J mice were collected and dissolved in 1× PBS, followed by low‐speed centrifugation to collect the supernatants. The supernatants were then anaerobically fermented in brain‐heart infusion broth medium with the addition of PBS, GLU, or GLU‐BA. Samples were collected at 0.5, 4, 8, 12, 24, 36, and 48 h for the determination of the total residual sugar, BA content, and microbial OD_600_.

### 4.8 Synthesis of ICG‐GLU‐BA

GLU was dissolved in isopropanol and stirred at room temperature for 1 h. Then, 10 mL of NaOH (20% w/w) was added and stirred for another hour. Afterward, a certain amount of chloroacetic acid was added, and the reaction proceeded at 60°C for 4 h. The pH was adjusted to 7.0, and the solution was dialyzed and lyophilized to obtain carboxylated GLU [58]. Subsequently, the carboxylated GLU was conjugated to the BA moiety, following the procedure described in the *Synthesis of GLU‐BA* section. Finally, the carboxylated GLU‐BA was dissolved in 2‐morpholinoethanesulphonic acid buffer solution, and 1‐(3‐dimethylaminopropyl)‐3‐ethylcarbodiimide and NH_2_‐ICG were added. The mixture was stirred at room temperature in the dark for 2 h. After the reaction, the solution was dialyzed until the dialysate became colorless and then freeze‐dried in the dark to obtain ICG‐GLU‐BA.

### 4.9 Biodistribution Analysis of GLU‐BA

ICG‐GLU‐BA (200 mg/kg) was orally administered to healthy male C57BL/6J mice. After oral administration, the mice were anchored on a flat platform and images were captured in the NIR‐II window (emission filter: 1100 nm long pass, excitation: 808 nm laser, exposure time: 10 ms) at predetermined time points using a commercial NIR‐II imaging system (DeepVision, Nirmidas Biotech Inc., USA).

### 4.10 Biocompatible Evaluation

For the acute biotoxicity study of GLU‐BA in vivo, we followed OECD Test Guideline 425 with minor modifications [33]. After 7 days of acclimatization, 12 mice were randomly divided into two groups (*n* = 6) and orally administered either 200 µL of PBS (PBS group) or GLU‐BA (850 mg/kg; GLU‐BA group) in a single dose on the first day. Mice were then continuously observed for variations in the body weight, abnormal behaviors (if any), death (if any), and food/water intake, and were then sacrificed after 14 days for further analysis.

For the sub‐chronic biotoxicity study of GLU‐BA in vivo, we followed OECD Test Guideline 407 with minor modifications [34]. After 7 days of acclimatization, 24 mice were randomly divided into four groups (*n* = 6) and orally administered 200 µL of PBS (PBS group), low‐dose GLU‐BA (10 mg/kg), medium‐dose GLU‐BA (50 mg/kg), and high‐dose GLU‐BA (250 mg/kg) daily for 28 consecutive days, respectively. Throughout the administration period, mice were continuously observed for variations in the body weight, abnormal behaviors (if any), death (if any), and food/water intake, and were then sacrificed after 28 days for further analysis.

For the long‐term biotoxicity study of GLU‐BA in vivo, we assessed the effects of GLU‐BA over a 366‐day period. After 7 days of acclimatization, 12 mice were randomly divided into two groups (*n* = 6) and orally administered either 200 µL of PBS (PBS group) or GLU‐BA (500 mg/kg; GLU‐BA group) on a daily basis for 366 consecutive days. During administration, mice were continuously observed for variations in the body weight, abnormal behaviors (if any), and death (if any), and were then sacrificed after 366 days for further analysis.

At the end of each experiment, blood samples were collected from each group for serum biochemistry analysis, including ALT, AST, TBIL, ALB, CREA, UA, BUN, and LDH. Major organs (heart, liver, spleen, lung, and kidney) were collected, weighed, sectioned, and stained with H&E for histopathological analysis.

### 4.11 Therapeutic Process of GLU‐BA Toward DSS‐Induced UC in Mice

The model establishment and sample collection processes were conducted as described in the *Metabolomics analysis of UC mice* section. Starting on day 8, to optimize the dosage of GLU‐BA in treating UC, 12 DSS‐induced UC mice were divided into four groups (*n* = 3) and orally administered low‐dose GLU‐BA (100 mg/kg), medium‐dose GLU‐BA (150 mg/kg), high‐dose GLU‐BA (200 mg/kg), or PBS (model group) daily for 6 consecutive days. Fecal consistency, fecal bleeding, and body weight changes were monitored daily throughout the experiment. Each parameter was scored according to previously proposed criteria [55] and used to calculate the DAI. At the end of the intervention, colonic tissues were collected for detailed analysis.

Starting on day 8, to evaluate the effectiveness of GLU‐BA in treating UC, 24 DSS‐induced UC mice were divided into four groups (*n* = 6) and orally administered GLU, GLU‐BA, 5‐ASA (positive control), or PBS daily for 6 consecutive days. Normal mice administered PBS orally were designated as the normal group. Fecal consistency, fecal bleeding, and body weight changes were monitored daily throughout the experiment. Each parameter was scored according to previously proposed criteria [55] and used to calculate the DAI. At the end of the intervention, colonic tissues were collected for detailed analysis, and feces were collected and stored at −80°C for subsequent analysis.

### 4.12 Therapeutic Process of GLU‐BA Toward DSS‐Induced Chronic UC in Mice

The modeling process followed a previous study [59]. C57BL/6J mice (male, 6–8 weeks old, 20 ± 0.2 g) were acclimatized for 7 days and then randomly assigned to different experimental groups. Chronic UC was induced through three repeated cycles of DSS administration. Each cycle consisted of exposure to 2.5% (w/v) DSS dissolved in drinking water for 5 days, followed by a 7‐day recovery period with normal drinking water. After completion of the third DSS cycle, mice were orally administered GLU‐BA or PBS once daily for 15 consecutive days. Body weight changes were monitored daily throughout the experiment. At the end of the intervention, colonic tissues were collected for detailed analysis.

### 4.13 Blockade of BA Interaction with GRP109A by MB in DSS‐Induced UC Mice

The model establishment and sample collection processes were conducted as described in the *Metabolomics analysis of UC mice* section. Starting on day 8, 10 UC mice were divided into two groups (*n* = 5) and orally administered GLU‐BA daily. Specifically, MB or saline was administered via intraperitoneal injection at a dosage of 5 mg/kg per day, 30 min before each GLU‐BA administration [41]. Fecal consistency, fecal bleeding, and body weight changes were monitored daily throughout the experiment. Each parameter was scored according to previously proposed criteria [55] and used to calculate the DAI. At the end of the intervention, colonic tissues were collected for detailed analysis.

### 4.14 Therapeutic Process of GLU‐BA Toward Cy‐Induced Immunosuppression in Mice

BALB/c mice (20 ± 0.2 g, female, 6–8 weeks) were acclimated for 7 days and then given intraperitoneal injections of Cy (80 mg/kg) for 3 consecutive days to establish an immunosuppression model, according to previous studies [51]. Starting on day 4, to evaluate the effectiveness of GLU‐BA in treating immunosuppression, 24 Cy‐induced immunosuppressive mice were divided into four groups (*n* = 6) and administered GLU, GLU‐BA, LH (positive control), or PBS daily for 7 consecutive days. Normal mice administered PBS orally were designated as the normal group. Body weight changes were monitored daily throughout the experiment. At the end of the intervention, small intestinal tissues were collected for detailed analysis, and feces were collected and stored at −80°C for subsequent analysis.

### 4.15 Determination of BA Concentration by GC

BA concentrations in gastrointestinal contents, fecal samples, and in vitro fermentation supernatants were determined by GC. Briefly, samples were acidified with 10% sulfuric acid and extracted twice with diethyl ether. The organic phases were combined, concentrated under a gentle stream of nitrogen, filtered through a 0.22 µm organic‐solvent‐compatible membrane, and subjected to GC analysis. GC analysis was performed using an Agilent 7890B GC equipped with a flame ionization detector (FID) and an HP‐INNOWAX capillary column (30 m × 0.25 mm × 0.25 µm, Agilent Technologies, USA). Helium was used as the carrier gas at a constant flow rate of 1.96 mL min^−1^. Samples (2 µL) were injected in the splitless mode. The injector and detector temperatures were maintained at 200°C and 250°C, respectively. The oven temperature was initially held at 75°C for 1 min, increased to 140°C at a rate of 6°C min^−1^, and then maintained at 140°C for 3 min.

### 4.16 16S rRNA Sequencing Measurement

Approximately 200 mg of fecal samples was added to PowerBead tubes and extracted using the DNeasy PowerSoil Kit (QIAGEN, Germany) according to the manufacturer's instructions. DNA purity and concentration were measured using a Nanodrop (NanoDrop Technologies, Wilmington, DE, USA) and Qubit 3.0 fluorometer (Life Technologies, Carlsbad, CA, USA). Sequencing libraries were constructed using Illumina's VAHTS DNA Adapters set3‐set6 (Vazyme Biotech, Nanjing, China), following the manufacturer's instructions with indexing codes. After quality assessment using a Qubit 3.0 fluorometer, sequencing was conducted.

### 4.17 Immunofluorescence Staining Analysis

Immunofluorescence staining was performed as previously described [60]. It was used to assess the expression levels of ZO‐1 and Occludin in the colon/small intestine and to characterize CD4^+^ and CD8^+^ T cells in the small intestine. After sequential incubation of tissue sections with primary antibodies (anti‐ZO‐1, anti‐Occludin, anti‐CD4, and anti‐CD8) and fluorescently labeled secondary antibodies (Alexa Fluor 488‐labelled anti‐IgG and Cy3‐labelled anti‐IgG), the levels of ZO‐1, Occludin, CD4, and CD8 were observed under a fluorescence microscope. The mean fluorescence density was assessed using ImageJ analysis software.

### 4.18 Enzyme Linked Immunosorbent Assay (ELISA)

Intestinal tissues were collected from the aforementioned therapeutic mice and analyzed for levels of various cytokines, including TNF‐α, IL‐6, TGF‐β, and IL‐10, as well as immunoglobulins such as IgA and IgG, using corresponding commercial ELISA kits. All procedures were performed in strict accordance with the manufacturer's instructions.

### 4.19 Statistical Analyses

Statistical analyses were performed using one‐way ANOVA and Student's t‐test. ns represents no significant difference, *< 0.05, **< 0.01, ***< 0.001, and ****< 0.0001 represent different levels of statistical significance.

## Author Contributions

**Feifei Xin**: methodology, software, data curation, validation, visualization, writing – original draft. **Zhike Xie**: methodology, software, data curation, formal analysis. **Danyue Zhao**: investigation, resources. **Lin Yang**: investigation, validation. **Yiqun Wan**: project administration. **Zuwei Liu**: methodology, formal analysis. **Situ Xiong**: software, resources. **Ying Zhang**: funding acquisition, visualization. **Lei Feng**: funding acquisition. **Hao Wan**: conceptualization, supervision, funding acquisition, project administration, writing – review and editing.

## Conflicts of Interest

The authors declare no conflicts of interest.

## Supporting information




**Supporting File**: advs77454‐sup‐0001‐SuppMat.docx.

## Data Availability

The data that support the findings of this study are available from the corresponding author upon reasonable request.
