# Supplementary material for: Oral Nanomicelle‐Mediated Sequential Butyrate Production in the Intestine for Ulcerative Colitis and Immunosuppression Treatment
Source: Adv Sci (Weinh). 2026 Aug 27:e77454. Online ahead of print. doi: 10.1002/advs.77454 (PMC13521179; doi:10.1002/advs.77454)
Supplement: Supplementary file 1 [file ADVS-9999-e77454-s001.docx]

Oral nanomicelle-mediated sequential butyrate production in the intestine for ulcerative colitis and immunosuppression treatment

Feifei Xin, Zhike Xie, Danyue Zhao, Lin Yang, Yiqun Wan, Zuwei Liu, Situ Xiong, Ying Zhang, Lei Feng & Hao Wan^*^

Feifei Xin, Zhike Xie, Lin Yang, Yiqun Wan, Zuwei Liu, Ying Zhang, Lei Feng and Hao Wan

State Key Laboratory of Food Science and Resources, Nanchang University, Nanchang 330047, Jiangxi, People’s Republic of China

**E-mail**: wanhao424@ncu.edu.cn; Hao Wan.

Danyue Zhao

Department of Food Science and Nutrition, The Hong Kong Polytechnic University, Hong Kong, People’s Republic of China;

Research Institute for Future Food, The Hong Kong Polytechnic University, Hong Kong, People’s Republic of China

Situ Xiong and Hao Wan

Department of Urology, The First Affiliated Hospital, Jiangxi Medical College, Nanchang University, Nanchang 330047, Jiangxi, People’s Republic of China

Supporting Information

**Tables**

**Table S1 |** Intestinal metabolomic analysis profiling the butyrate (BA) contents in ulcerative colitis (UC) patients and healthy individuals from previous studies.

| **Article title/**  **Reference** | **Number of UC patients** | **Number of healthy** **individuals** | **Determination method** | **BA contents in UC patients (mM)** | **BA contents in healthy individuals (mM)** | **BA contents (UC vs healthy)** | ***p* value** |
| --- | --- | --- | --- | --- | --- | --- | --- |
| Imbalance in intestinal microflora constitution could be involved in the pathogenesis of inflammatory bowel disease/ ^[1]^ | 39 | 10 | High-performance liquid chromatography | 8.9 ± 8.1 | 15.9 ± 8.6 | Significantly decreased | < 0.05 |
| Fecal Lactate and Ulcerative Colitis/ ^[2]^ | 20 (Severe UC) | 29 | Gas-liquid chromatography | 4.3 ± 5.0 | 13.7 ± 8.4 | Significantly decreased | < 0.001 |

**Table S2 |** Analytical validation of the gas chromatography method for BA quantification.

| **Parameter** | **Result** |
| --- | --- |
| Linear range (mmol/L) | 0.005-70 |
| Regression equation | y = 1901x-364 |
| Correlation coefficient (R²) | 0.9997 |
| Intra-day precision (RSD, %) | 0.13%-0.37% |
| Inter-day precision (RSD, %) | 0.04%-0.11% |
| Recovery (%) | 98.1%-102.9% |

**Table S3 |** Summary of literature reporting intestinal supplementation of therapeutic components that adopted either burst-release or gradual-production mode and their corresponding therapeutic effects toward UC in mice.

| **Article title/**  **Reference** | **Supplementation mode** | **Therapeutic component** | **Figure** | **Therapeutic effect** |
| --- | --- | --- | --- | --- |
| An Engineered Butyrate-Derived Polymer Nanoplatform as a Mucosa-Healing Enhancer Potentiates the Therapeutic Effect of Magnolol in Inflammatory Bowel Disease/ ^[3]^ | Gradual supplementation | Magnolol (Mag) |  | Daily PSBA@Mag treatment for consecutive 9 days prevented body weight loss progression. |
| Magnolol-loaded polydopamine-chitosan coated pH-responsive nanoparticles for alleviation of ulcerative colitis/ ^[4]^ | Gradual supplementation | Mag |  | The weight loss induced by dextrose sodium sulfate (DSS) treatment was inhibited by some extent in mice treated with either MAG or MZPcc. |
| Nano-in-micro alginate/chitosan hydrogel via electrospray technology for orally curcumin delivery to effectively alleviate ulcerative colitis/ ^[5]^ | Gradual supplementation | Curcumin |  | Oral-Cur@NMPs or HA-Zein/Cur nanoparticles were able to slow the weight loss progression caused by DSS. |
| Construction of Chitosan/Alginate Nano-Drug Delivery System for Improving Dextran Sodium Sulfate-Induced Colitis in Mice/ ^[6]^ | Gradual supplementation | 3, 5, 4’ -trihydroxystilbene (RES) |  | It was found that oral administration of RES-PCAC-NPs could effectively alleviate weight loss. |
| Orally deliverable sequence-targeted astaxanthin nanoparticles for colitis alleviation/ ^[7]^ | Gradual supplementation | Astaxanthin (ASX) |  | After treatment for 7 days, ASX-based nanoparticles began to inhibit the continuous weight loss. |
| Oral chitosan-cyclodextrin “shell-core” nanoparticles co-loaded Rhein and chlorogenic acid for ulcerative colitis treatment/ ^[8]^ | Burst release | Rhein (RH) and chlorogenic acid (CGA) |  | Upon oral administration of CP@CGA-FA/TA@RH once a day (the gavage dosage was above 300 mg/kg) for continuous 7 days, the nanomaterials could alleviate the weight loss of mice caused by DSS. |
| Pectin-loaded ferulic acid nanoparticles: A potential therapeutic strategy for ulcerative colitis via modulation of the cGAS-STING pathway/ ^[9]^ | Burst release | Ferulic acid |  | After gavage once a day for 7 consecutive days, PC-FA (300 mg/kg) significantly mitigated weight loss, and the higher-dose PC-FA group (600 mg/kg) showed an increasing trend for the body weight. |
| Treatment of peanut allergy and colitis in mice via the intestinal release of butyrate from polymeric micelles/ ^[10]^ | Burst release | BA |  | Gavage of low (200 mg/kg), medium (400 mg/kg), or high (800 mg/kg) dosages twice daily for continuous 10 days reduced the concentration of orally administered FITC-dextran in blood, indicating the repairment of the intestinal barrier. |
| The investigation on sialic acid-modified pectin nanoparticles loaded with oxymatrine for orally targeting and inhibiting the of ulcerative colitis/ ^[11]^ | Burst release | Oxymatrine (OMT) |  | Oral administration of OMT/SA-NPs (1200 mg/kg/day for 7 consecutive days) prevented DSS-induced body weight loss in mice. |
| Polyvinyl Butyrate Nanoparticles as Butyrate Donors for Colitis Treatment/ ^[12]^ | Burst release | BA |  | Daily oral administration of low (414 mg/kg) or high dosages (837 mg/kg) of NP for continuous 10 days mitigated weight loss induced by DSS. |
| Oral nanomicelle-mediated sequential in-situ butyrate production in the intestine for ulcerative colitis and immunosuppression treatment/ Our study | Burst yet gradual production | BA |  | Our strategy required only 150 mg/kg per day to achieve body weight recovery within only 6 days. |

**Table S4 |** Summary of literature reporting intestinal supplementation of therapeutic components and their corresponding therapeutic effects toward cyclophosphamide (Cy)-induced immunosuppression in mice.

| **Article title/**  **Reference** | **Therapeutic components** | **Figure** | **Therapeutic effect** |
| --- | --- | --- | --- |
| Immunomodulatory activity of low molecular-weight peptides from *Nibea japonica* skin in cyclophosphamide-induced immunosuppressed mice/ ^[13]^ | Peptides from *Nibea japonica* skin |  | After 21 consecutive days of daily oral administration of NJSP at a dosage of 400 mg/kg, the sharp body weight loss caused by Cy-induced immunosuppression was attenuated by some extent. |
| Immunoenhancement effect of crude polysaccharides of *Helvella leucopus* on cyclophosphamide-induced immunosuppressive mice/ ^[14]^ | Polysaccharides of *Helvella leucopus* (HLP) |  | Compared to the model group (CTX), treatment with HLP (daily dosage of 200 mg/kg) only resulted in a slight increase in the body weight after 14 consecutive days. |
| Immunomodulatory activity of a water-soluble polysaccharide extracted from mussel on cyclophosphamide-induced immunosuppressive mice models/ ^[15]^ | Polysaccharide extracted from mussel |  | Body weight of the Cy group gradually decreased after Cy administration, whereas Cy + LMP (300 mg/kg/day for 21 days) and Cy + HMP (600 mg/kg/day for 21 days) treatments inhibited weight loss in mice. |
| The Immuno-Enhancement Effects of Tubiechong (*Eupolyphaga sinensis*) Lyophilized Powder in Cyclophosphamide-Induced Immunosuppressed Mice/ ^[16]^ | Tubiechong (*Eupolyphaga sinensis*) lyophilized powder |  | Compared with the normal group (CS), the thymus index was significantly decreased in the CTX-treatment group (MS), but increased after 14-day consecutive treatment with a medium dosage of ESL (ESL-M; 1000 mg/kg/day). |
| Protective effect of centella triterpene saponins against cyclophosphamide-induced immune and hepatic system dysfunction in rats: its possible mechanisms of action/ ^[17]^ | Centella triterpene saponins (EXT) |  | CYP treatment drastically reduced the spleen weight, whereas 30-day consecutive administration of EXT (250 mg/kg/day) increased the spleen weight. |
| Ginger polysaccharides enhance intestinal immunity by modulating gut microbiota in cyclophosphamide-induced immunosuppressed mice/ ^[18]^ | Ginger polysaccharides UGP1 |  | UGP1 (group H; 300 mg/kg/day for 13 consecutive days) significantly alleviated CTX-induced body weight loss. |
| Immuno-stimulatory activity of *Astragalus* polysaccharides in cyclophosphamide-induced immunosuppressed mice by regulating gut microbiota/ ^[19]^ | *Astragalus* polysaccharides (APS) |  | CTX administration induced significant body weight loss in mice, which was not significantly ameliorated by APS (200 mg/kg/day for 10 consecutive days) treatment. |
| Oral nanomicelle-mediated sequential in-situ butyrate production in the intestine for ulcerative colitis and immunosuppression treatment/ Our study | BA |  | Our strategy only required a dosage of 150 mg/kg per day to achieve body weight recovery in Cy-induced immunosuppressive mice within 7 days. |

**Figures**

**Figure S1 |** Food (**a**) and water (**b**) intake of normal and UC mice.

**Figure S2 |** Colon photographs (**a**) and corresponding quantified lengths (**b**) of normal and UC mice, *n* = 12 per group. Statistical analyses were performed using Student's t-test. **** < 0.0001 represents a statistical significance.

**Figure S3 |** Representative hematoxylin and eosin (H&E) staining images of colon tissues harvested from normal and UC mice.

**Figure S4 |** Quality control (QC) correlation plots for metabolomics analysis of normal and UC mice.

**Figure S5 |** Colon photographs of mice from the normal, model and sodium butyrate (NaBA) groups, *n* = 10 per group.

**Figure S6 |** Gastrointestinal distribution of BA after oral administration of NaBA in the DSS-induced UC mouse model. (**a**) BA levels within the stomach at 0.5 h post oral administration of PBS or NaBA. (**b-d**) BA levels within the intestine at 12 h post oral administration of PBS or NaBA, including ileum (**b**), cecum (**c**), and colon (**d**). Statistical analyses were performed using Student's t-test. ns represents no significance and ** < 0.01 represents a statistical significance.

**Figure S7 |** Synthetic scheme of the β-glucan-butyrate (GLU-BA) macromolecule.

**Figure S8 |** ^1^H nuclear magnetic resonance (NMR) spectrum of GLU.

**Figure S9 |** ^1^H NMR spectrum of GLU-BA.

**Figure S10 |** High-performance gel permeation chromatography (HPGPC) calibration curve using dextran and glucose as the standards.

Figure **S11 |** Particle size variation of GLU-BA nanomicelles during simulated gastrointestinal digestion, *n* = 3 per group.

**Figure S12 |** Variation of total residual sugar amounts from 0-4 h in the microbial fermentation assay, *n* = 3 per group. Statistical analyses were performed using one-way analysis of variance (ANOVA). *** < 0.05, ** < 0.01, and *** < 0.001 represent different levels of statistical significance compared with the phosphate buffered saline (PBS) group.

**Figure S13 |** Variation of BA contents from 0-4 h in the microbial fermentation assay, *n* = 3 per group. Statistical analyses were performed using ANOVA. ns represents not significant compared with the PBS group. *** < 0.001 and **** < 0.0001 represent different levels of statistical significance.

**Figure S14 |** Bacterial growth curves during *in vitro* fermentation assay from 0-4 h. Bacterial growth was measured by absorbance at 600 nm (i.e., optical density at 600 nm [OD_600_]), *n* = 3 per group. Statistical analyses were performed using ANOVA. ns represents not significant compared with the PBS group. *** < 0.001 and **** < 0.0001 represent different levels of statistical significance.

**Figure S15 |** Second near-infrared (NIR-II) fluorescence imaging (long-pass filter: 1100 nm; laser: 808 nm; exposure time: 10 ms) of indocyanine green fluorophore-labelled GLU-BA (ICG-GLU-BA).

**Figure S16 |** Food (**a**) and water (**b**) intake of mice in the PBS and GLU-BA groups during the acute toxicity assessment*.*

**Figure S17 |** Liver function analysis of mice from the PBS and GLU-BA groups, including alanine aminotransferase (ALT; **a**), aspartate transaminase (AST; **b**), total bilirubin (TBIL; **c**), and albumin (ALB; **d**), *n* = 6 per group. Statistical analyses were performed using Student's t-test. ns represents no significance.

**Figure S18 |** Kidney function analysis of mice from the PBS and GLU-BA groups, including creatinine (CREA; **a**), blood urea nitrogen (BUN; **b**), and uric acid (UA; **c**), *n* = 6 per group. Statistical analyses were performed using Student's t-test. ns represents no significance.

**Figure S19 |** Lactate dehydrogenase (LDH) analysis of mice from the PBS and GLU-BA groups, *n* = 6 per group. Statistical analyses were performed using Student's t-test. ns represents no significance.

**Figure S20 |** Food (**a**) and water (**b**) intake of mice in the PBS, low-dose, medium-dose, and high-dose GLU-BA groups during the sub-chronic toxicity assessment.

**Figure S21 |** Organ weight of mice from the PBS, low-dose, medium-dose, and high-dose GLU-BA groups, including heart (**a**), liver (**b**), spleen (**c**), lung (**d**), and kidney (**e**). Statistical analyses were performed using one-way ANOVA. ns represents no significance.

**Figure S22 |** Liver function analysis of mice from the PBS and GLU-BA groups, including ALT (**a**) and AST (**b**). Kidney function analysis of mice from the PBS and GLU-BA groups, including BUN (**c**) and CREA (**d**), *n* = 6 per group. Statistical analyses were performed using Student's t-test. ns represents no significance.

**Figure S23 |** Representative H&E staining images of colonic tissues harvested from the PBS and GLU-BA groups at the endpoint of long-term toxicity evaluation study.

**Figure S24 | a,** C57BL/6J mice were given drinking water containing 3% DSS continuously for 7 consecutive days to establish a UC mouse model. Afterward, PBS, a low dose of GLU-BA (100 mg/kg), a medium dose of GLU-BA (150 mg/kg), or a high dose of GLU-BA (200 mg/kg) was given orally daily for 6 consecutive days. **b,** Changes in the body weight of mice over time in different groups, *n* = 3 per group. **c,** Changes in disease activity index (DAI) of mice over time in different groups, *n* = 3 per group. Statistical analyses were performed using one-way ANOVA. ns represents no significance. *< 0.05, **< 0.01, and ***< 0.001 represent different statistical significances.

**Figure S25 | a,** Representative images of colons harvested from mice across different groups after therapeutic interventions, *n* = 3 per group. **b,** Quantification of colon length of mice from different groups after therapeutic interventions, *n* = 3 per group. PBS-treated, GLU-BA (low dose)-treated, GLU-BA (medium dose)-treated, and GLU-BA (high dose)-treated mice were labeled as “Model”, “Low”, “Medium”, and “High”, respectively, in the figure. Statistical analyses were performed using one-way ANOVA. ns represents no significance. **< 0.01 and ***< 0.001 represent different statistical significances.

**Figure S26 |** Spleen indices of mice from different groups after therapeutic interventions, *n* = 3 per group. PBS-treated, GLU-BA (low dose)-treated, GLU-BA (medium dose)-treated, and GLU-BA (high dose)-treated mice were labeled as “Model”, “Low”, “Medium”, and “High”, respectively, in the figure. Statistical analyses were performed using one-way ANOVA. ns represents no significance. **< 0.01 and ***< 0.001 represent different statistical significances.

**Figure S27 |** Representative H&E staining images of colonic tissues harvested from mice across different groups after therapeutic interventions.

**Figure S28 | Comparison of the therapeutic efficacy of GLU-BA and GLU+BA in the DSS-induced UC mouse model. a,** C57BL/6J mice were given drinking water containing 3% DSS continuously for 7 consecutive days to establish a UC mouse model. Afterward, GLU-BA or GLU+BA was given orally administration daily for 6 consecutive days. **b**, Changes in the body weight of mice over time in different groups, *n* = 6 per group. **c**, Changes in DAI of mice over time in different groups, *n* = 6 per group. **d**, Representative images of colons harvested from mice across different groups after therapeutic interventions, *n* = 6 per group. **e**, Quantification of colon length of mice from different groups after therapeutic interventions, *n* = 6 per group. **f**, Spleen index of mice from different groups after therapeutic interventions, *n* = 6 per group. **g**, Representative H&E and alcian blue-periodic acid-schiff (AB-PAS) staining images of colonic tissues harvested from mice across different groups after therapeutic interventions. Statistical analyses were performed using Student's t-test. *< 0.05, **< 0.01, and ***< 0.001 represent different statistical significances.

**Figure S29 | Therapeutic efficacy of GLU-BA in the DSS-induced chronic UC mouse model.** **a,** Schematic illustration of the establishment of a DSS-induced chronic UC mouse model. C57BL/6J mice were treated with three cycles of DSS exposure (2.5% DSS) and recovery periods over 36 days to induce chronic UC. After model establishment, mice received oral administration of GLU-BA, and samples were collected for evaluation of therapeutic efficacy. **b,** Changes in the body weight of mice over time in different groups, *n* = 6 per group. **c,** Representative images of colons harvested from mice across different groups after therapeutic interventions, *n* = 6 per group. **d,** Quantification of colon length of mice from different groups after therapeutic interventions, *n* = 6 per group. **e,** Spleen index of mice from different groups after therapeutic interventions, *n* = 6 per group. **f,** Representative H&E and AB-PAS staining images of colonic tissues harvested from mice across different groups after therapeutic interventions. Statistical analyses were performed using Student's t-test. **< 0.01 represents statistical significance.

**Figure S30 | Validation of the therapeutic efficacy of MB in the DSS-induced UC mouse model. a,** C57BL/6J mice were given drinking water containing 3% DSS continuously for 7 consecutive days to establish a UC mouse model. Afterward, MB or saline was administered by intraperitoneal injection daily for 6 consecutive days. **b,** Changes in the body weight of mice over time in different groups, *n* = 6 per group. **c,** Changes in DAI of mice over time in different groups, *n* = 6 per group. **d,** Representative images of colons harvested from mice across different groups after therapeutic interventions, *n* = 6 per group. **e,** Quantification of colon length of mice from different groups after therapeutic interventions, *n* = 6 per group. **f,** Spleen index of mice from different groups after therapeutic interventions, *n* = 6 per group. **g**, Representative H&E and AB-PAS staining images of colonic tissues harvested from mice across different groups after therapeutic interventions. Statistical analyses were performed using Student's t-test. ns represents no significance.

**Figure S31 |** Microbial communities of the normal, model, GLU, and GLU-BA groups in the UC treatment application, as analyzed by Shannon index curve, *n* = 6 per group.

**Figure S32 |** β-diversity of the gut microbiota of the normal, model, GLU, and GLU-BA groups in the UC treatment application, as analyzed according to the principal component analysis (PCA) based on the Bray-Curtis distance, *n* = 6 per group.

**Figure S33 |** Relative abundance (%) of *Proteobacteria* in the normal, model, GLU, and GLU-BA groups in the UC treatment application, *n* = 6 per group. Statistical analyses were performed using one-way ANOVA. * < 0.05 represents a statistical significance.

**Figure S34 |** Relative abundance (%) of *Bacteroidia* in the normal, model, GLU, and GLU-BA groups in the UC treatment application, *n* = 6 per group. Statistical analyses were performed using one-way ANOVA. ns represents not significant. ** < 0.01 and ***< 0.001 represent different statistical significances.

**Figure S35 |** Relative abundance (%) of *Escherichia-Shigella* in the normal, model, GLU, and GLU-BA groups in the UC treatment application, *n* = 6 per group. Statistical analyses were performed using one-way ANOVA. * < 0.05 represents a statistical significance.

**Figure S36 |** Food (**a**) and water (**b**) intake of mice from the normal, model, GLU, GLU-BA, and LH groups in the immunosuppression treatment application.

**Figure S37 |** Representative AB-PAS staining images of small intestines in immunosuppressive mice from the model and GLU-BA groups.

**Figure S38 |** Representative immunofluorescence staining images showing ZO-1 protein expression in immunosuppressive mice from the model and GLU-BA groups. The nucleus was stained with DAPI.

**Figure S39 |** Representative immunofluorescence staining images showing Occludin protein expression in immunosuppressive mice from the model and GLU-BA groups. The nucleus was stained with DAPI.

**Figure S40 |** Microbial communities of Cy-induced immunosuppressive mice from the model and GLU-BA groups, as analyzed by the Shannon index curve, *n* = 5 per group.

**Figure S41 |** α-diversity of the gut microbiota of the model and GLU-BA groups in the immunosuppression treatment application, as analyzed according to the Chao1, *n* = 5 per group. Statistical analyses were performed using Student's t-test. * < 0.05 represents a statistical significance.

**Figure S42 |** β-diversity of mice from the model and GLU-BA groups in the immunosuppression treatment application, as analyzed according to the PCA based on the Bray-Curtis distance, *n* = 5 per group.

**Figure S43 |** Relative abundance (%) of *Proteobacteria* in the model and GLU-BA groups in the immunosuppression treatment application, *n* = 5 per group. Statistical analyses were performed using Student's t-test. * < 0.05 represents a statistical significance.

**Figure S44 |** Relative abundance (%) of *Gammaproteobacteria* in the model and GLU-BA groups in the immunosuppression treatment application, *n* = 5 per group. Statistical analyses were performed using Student's t-test. * < 0.05 represents a statistical significance.

**References**

1.	H. Takaishi, T. Matsuki, A. Nakazawa, T. Takada, S. Kado, T. Asahara et al., "Imbalance in intestinal microflora constitution could be involved in the pathogenesis of inflammatory bowel disease," *Int. J. Med. Microbiol.* (2008): 298, 463-472, https://doi.org/10.1016/j.ijmm.2007.07.016.

2.	P. Vernia, R. Caprilli, G. Latella, F. Barbetti, F.M. Magliocca and M. Cittadini "Fecal Lactate and Ulcerative Colitis," *Gastroenterology* (1988): 95, 1564-1568, https://doi.org/10.1016/S0016-5085(88)80078-7.

3.	X. Fan, Z. Zhang, W. Gao, Q. Pan, K. Luo, B. He et al., "An Engineered Butyrate-Derived Polymer Nanoplatform as a Mucosa-Healing Enhancer Potentiates the Therapeutic Effect of Magnolol in Inflammatory Bowel Disease," *ACS Nano* (2024): 18, 229-244, https://doi.org/10.1021/acsnano.3c05732.

4.	G. Mu, F. Peng, Z. Zhang, J.M. Brameld, P. Rose, X. Yang et al., "Magnolol-loaded polydopamine-chitosan coated pH-responsive nanoparticles for alleviation of ulcerative colitis," *J. Drug Delivery Sci. Technol.* (2024): 101, 106151, https://doi.org/10.1016/j.jddst.2024.106151.

5.	C. Zhang, X. Wang, M. Xiao, J. Ma, Y. Qu, L. Zou et al., "Nano-in-micro alginate/chitosan hydrogel via electrospray technology for orally curcumin delivery to effectively alleviate ulcerative colitis," *Mater. Des.* (2022): 221, 110894, https://doi.org/10.1016/j.matdes.2022.110894.

6.	M. Jin, S. Li, Y. Wu, D. Li and Y. Han "Construction of Chitosan/Alginate Nano-Drug Delivery System for Improving Dextran Sodium Sulfate-Induced Colitis in Mice," *Nanomaterials (Basel)* (2021): 11, 1884, https://doi.org/10.3390/nano11081884.

7.	Y. Chen, W. Su, S. Tie, W. Cui, X. Yu, L. Zhang et al., "Orally deliverable sequence-targeted astaxanthin nanoparticles for colitis alleviation," *Biomaterials* (2023): 293, 121976, https://doi.org/10.1016/j.biomaterials.2022.121976.

8.	W. Nie, W. Zhong, L. Qian, H. Zhong, Y. Hou, H. Xu et al., "Oral chitosan-cyclodextrin “shell-core” nanoparticles co-loaded Rhein and chlorogenic acid for ulcerative colitis treatment," *Int. J. Biol. Macromol.* (2025): 288, 138493, https://doi.org/10.1016/j.ijbiomac.2024.138493.

9.	J. Xiao, S. Zhou, F. Fei, L. Long and C. Guo "Pectin-loaded ferulic acid nanoparticles: A potential therapeutic strategy for ulcerative colitis via modulation of the cGAS-STING pathway," *Toxicol. Appl. Pharmacol.* (2025): 499, 117317, https://doi.org/10.1016/j.taap.2025.117317.

10.	R. Wang, S. Cao, M.E.H. Bashir, L.A. Hesser, Y. Su, S.M.C. Hong et al., "Treatment of peanut allergy and colitis in mice via the intestinal release of butyrate from polymeric micelles," *Nat. Biomed. Eng.* (2023): 7, 38-55, https://doi.org/10.1038/s41551-022-00972-5.

11.	C. Zhao, X. Yang, M. Fan, L. Tian, T. Sun, C. Sun et al., "The investigation on sialic acid-modified pectin nanoparticles loaded with oxymatrine for orally targeting and inhibiting the of ulcerative colitis," *Colloids Surf., B* (2024): 236, 113809, https://doi.org/10.1016/j.colsurfb.2024.113809.

12.	Y. Mu, Y. Kinashi, J. Li, T. Yoshikawa, A. Kishimura, M. Tanaka et al., "Polyvinyl Butyrate Nanoparticles as Butyrate Donors for Colitis Treatment," *ACS Appl. Bio Mater.* (2021): 4, 2335-2341, https://doi.org/10.1021/acsabm.0c01105.

13.	F. Yu, K. He, X. Dong, Z. Zhang, F. Wang, Y. Tang et al., "Immunomodulatory activity of low molecular-weight peptides from Nibea japonica skin in cyclophosphamide-induced immunosuppressed mice," *J. Funct. Foods* (2020): 68, 103888, https://doi.org/10.1016/j.jff.2020.103888.

14.	W.-N. Zhang, L.-L. Gong, Y. Liu, Z.-B. Zhou, C.-X. Wan, J.-J. Xu et al., "Immunoenhancement effect of crude polysaccharides of Helvella leucopus on cyclophosphamide-induced immunosuppressive mice," *J. Funct. Foods* (2020): 69, 103942, https://doi.org/10.1016/j.jff.2020.103942.

15.	X. Xiang, R. Wang, L. Chen, Y. Chen, B. Zheng, S. Deng et al., "Immunomodulatory activity of a water-soluble polysaccharide extracted from mussel on cyclophosphamide-induced immunosuppressive mice models," *npj Sci. Food* (2022): 6, 26, https://doi.org/10.1038/s41538-022-00140-8.

16.	H.-H. Liu, Y.-l. Yan, F.-l. Zhang and Q.-f.J.I.I. Wu "The Immuno-Enhancement Effects of Tubiechong (Eupolyphaga sinensis) Lyophilized Powder in Cyclophosphamide-Induced Immunosuppressed Mice," *Immunol. Invest.* (2019): 48, 844-859, https://doi.org/10.1080/08820139.2019.1588291.

17.	P. Duggina, C.M. Kalla, S.R. Varikasuvu, S. Bukke and V. Tartte "Protective effect of centella triterpene saponins against cyclophosphamide-induced immune and hepatic system dysfunction in rats: its possible mechanisms of action," *J. Physiol. Biochem.* (2015): 71, 435-454, https://doi.org/10.1007/s13105-015-0423-y.

18.	J.-p. Liu, J. Wang, S.-x. Zhou, D.-c. Huang, G.-h. Qi and G.-t. Chen "Ginger polysaccharides enhance intestinal immunity by modulating gut microbiota in cyclophosphamide-induced immunosuppressed mice," *Int. J. Biol. Macromol.* (2022): 223, 1308-1319, https://doi.org/10.1016/j.ijbiomac.2022.11.104.

19.	Y. Li, J. Zheng, Y. Wang, H. Yang, L. Cao, S. Gan et al., "Immuno-stimulatory activity of Astragalus polysaccharides in cyclophosphamide-induced immunosuppressed mice by regulating gut microbiota," *Int. J. Biol. Macromol.* (2023): 242, 124789, https://doi.org/10.1016/j.ijbiomac.2023.124789.
